# Unraveling Interband Hot‐Electron Transfer in Hydrogenated Au@Cu_2_O/TiO_2_ Heterostructure Nanocrystals for Enhanced Hydrogen Evolution

**DOI:** 10.1002/smll.202511114

**Published:** 2026-02-23

**Authors:** Tsai‐Te Wang, Shan‐Jen Yang, Sudhakar Narra, Rohit R Koli, Yu‐Ru Lin, Yi‐Dong Lin, Eric Wei‐Guang Diau, Yung‐Jung Hsu, Yan‐Gu Lin, Ming‐Chang Lin

**Affiliations:** ^1^ Scientific Research Division National Synchrotron Radiation Research Center Hsinchu Taiwan; ^2^ Department of Materials Science and Engineering National Yang Ming Chiao Tung University Hsinchu Taiwan; ^3^ Department of Applied Chemistry National Yang Ming Chiao Tung University Hsinchu Taiwan; ^4^ Center for Emergent Functional Matter Science National Yang Ming Chiao Tung University Hsinchu Taiwan

**Keywords:** Au@Cu_2_O Core–shell, heterostructure, hydrogen, photocatalysis, synergistic, TiO_2_

## Abstract

We report the design and fabrication of a novel hydrogenated photoactive composite, H:(Au@Cu_2_O/TiO_2_). Transmission electron microscopy (TEM) and X‐ray diffraction (XRD) confirmed a well‐defined core–shell architecture with a *p–n* heterojunction configuration. Optical and electronic band structures were systematically investigated. The localized surface plasmon resonance LSPR‐induced inter‐band electron transfer process from Au to Cu_2_O shell was elucidated from the transient absorption spectra (TAS) and in situ X‐ray absorption spectroscopy (XAS). A comprehensive charge‐transfer mechanism was provided; the Z‐scheme configuration facilitates charge transfer and separation for improved efficiency with Cu_2_O shell and TiO_2_ NPs to complete the redox cycle. The hydrogenated heterostructure exhibited an exceptional H_2_ evolution rate of 9.3 mmol g^−^^1^ h^−^^1^ under AM 1.5 illumination while maintaining excellent stability. This enhancement is mainly attributed to direct injection of Au LSPR, a 3.9 times improvement with respect to H:(Cu_2_O/TiO_2_) of 2.4 mmol g^−1^ h^−1^ which was not reported before. Apparent quantum yield (AQY) measurements reached 2.5% at 650 nm and 0.8% at 800 nm, effectively extending the photocatalytic activity into the near‐infrared region. These results highlight the robust H:(Au@Cu_2_O/TiO_2_) photocatalyst as a highly promising platform for photocatalytic hydrogen evolution with methanol as an efficient hole scavenger and broader photoconversion applications.

## Graphical abstract

Hydrogenated Au@Cu_2_O/TiO_2_ nanocrystals with oxygen‐vacancy‐engineered heterostructures enable efficient hot‐electron transfer. A Z‐scheme heterojunction forms between TiO_2_ and Cu_2_O, suppressing electron‐hole recombination and leading to a hydrogen evolution rate of 9.3 mmol g^−^^1^ h^−^^1^, with AQYs of 2.5% at 650 nm and 0.8 % at 800 nm, extending activity into the near‐infrared region.

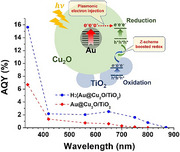

## 1 Introduction

Conventional fossil fuel reserves are finite and also contribute significantly to environmental degradation through carbon dioxide emissions. In contrast, hydrogen represents a clean, renewable, and emission‐free energy alternative. However, H_2_ is not naturally abundant. A three‐color model has been designed to classify the method and energy adopted in hydrogen production [1, 2]. The gray type hydrogen was obtained from fossil fuel steam reforming with highly pollutant emission, the controlled emission with carbon capture and storage technology was referred to as the blue type hydrogen. The production of green type hydrogen was considered to be environment friendly by *a clean, renewable, and emission‐free energy alternative* scenario. Herein, enhancing the efficiency of solar‐driven hydrogen production can help stabilize energy supply, promote the adoption of renewable energy sources, mitigate environmental pollution [3], and reduce the emission of harmful gases [4, 5]. The mechanism of photocatalysts’ energy transfer processes may be provided by *(i) photo‐excited separation of electron and hole pair, (ii) transportation to the reaction part, (iii) completion of the redox cycle by hydrogen and oxygen generation* [6]. Thus, the strategy for developing efficient photocatalytic materials involves critical concepts was described briefly: the narrow bandgap of a photocatalysts is expected to increase the amount of electron‐hole pairs under solar illumination; Materials with good band alignment can help suppress charge recombination and success charge transfer.

Cu_2_O substrates have attracted great interest for their narrow bandgaps and high photo‐sensitivity for application in the visible region of the solar radiation. The recombination of photo‐excited electron and hole pair has been a serious concern in Cu_2_O modification, such as decorated by particles [7], doping [8], and effective heterojunction [9] with respect to the Cu_2_O *p‐*type characteristics. Core–shell structured materials have attracted considerable attention due to their nontoxic nature, ease of synthesis, and scalability [10, 11]. These hybrid heterostructures, composed of a metallic core and a semiconductor shell, exhibit favorable band alignments that enhance interfacial charge transport and suppress the recombination of photoexcited charge carriers, thereby improving photocatalytic performance [11, 12]. In particular, the precisely engineered Au@Cu_2_O structure by controlling Au core size and Cu_2_O shell thickness [12] optimized the localized surface plasmon resonance (SPR) from the gold core to generate high‐energy hot electrons. These electrons are injected into the conduction band (CB) of the Cu_2_O shell via a direct electron transfer (DET) mechanism, providing additional charge carriers for enhanced photocatalysis [10, 13, 14, 15]. Moreover, the *p‐*type semiconductor characteristics of Cu_2_O can be coupled with various *n*‐type semiconductors—such as CdS [16, 17], ZnO [17, 18], TiO_2_ [19, 20] and Ga_2_O_3_ [21, 22]—to form *p–n* heterojunctions with suitable band alignments that promote the Fermi level equilibration. The built‐in electric field at the heterojunction interface facilitates efficient separation of photoinduced electron–hole pairs. Simultaneously, the TiO_2_ photoexcited electrons were transferred to Cu_2_O valence band in combination with Cu_2_O photogenerated holes following the Z‐scheme structure, leading to the reduced e‐h recombination and thus boosting the photoactivity. Herein, the electrons are preserved on Cu_2_O for efficient reduction, and the photogenerated holes are accumulated on the TiO_2_ surface, enabling oxidation reactions and completing the redox cycle [11, 15]. Specifically, the excited electrons may be trapped in the defect sites created by the hydrogenation effect [23, 24], resulting in hydrogen generation enhancement as demonstrated well in our earlier works [23, 24].

In this study, a novel hydrogenated heterostructure composite, denoted as H:(Au@Cu_2_O/TiO_2_), was rationally designed. Building upon the Au@Cu_2_O core–shell framework, TiO_2_ nanoparticles were uniformly anchored onto the Cu_2_O shell. The structural and optical characteristics of the composite were systematically investigated using scanning electron microscopy (SEM), transmission electron microscopy (TEM), X‐ray diffraction (XRD), UV–Vis spectroscopy, and Raman spectroscopy. Time‐resolved photoluminescence (TRPL) was employed to probe charge carrier dynamics at the interface. As illustrated in the table of content (TOC), the construction of photoexcited catalysts H:(Au@Cu_2_O/TiO_2_) was presented. SPR excitation in the Au core generates energetic hot electrons, which are transferred to the Cu_2_O shell. Transient absorption spectra (TAS) were employed for the fast inter‐band transition studies. The heterojunction formed between *p‐*type Cu_2_O and *n*‐type TiO_2_ enhances charge separation, facilitating efficient hydrogen evolution on the Cu_2_O surface and methanol oxidation on the TiO_2_ side. Photoelectrochemical (PEC) measurements provide solid information on the spatially separated redox site. The H:(Au@Cu_2_O/TiO_2_) composite was synthesized via hydrogenation in methanol vapor [23, 24], which also increased the density of oxygen vacancies. The results obtained from this systematic study are reported herein.

## 2 Results and Discussion

**FIGURE 1:**
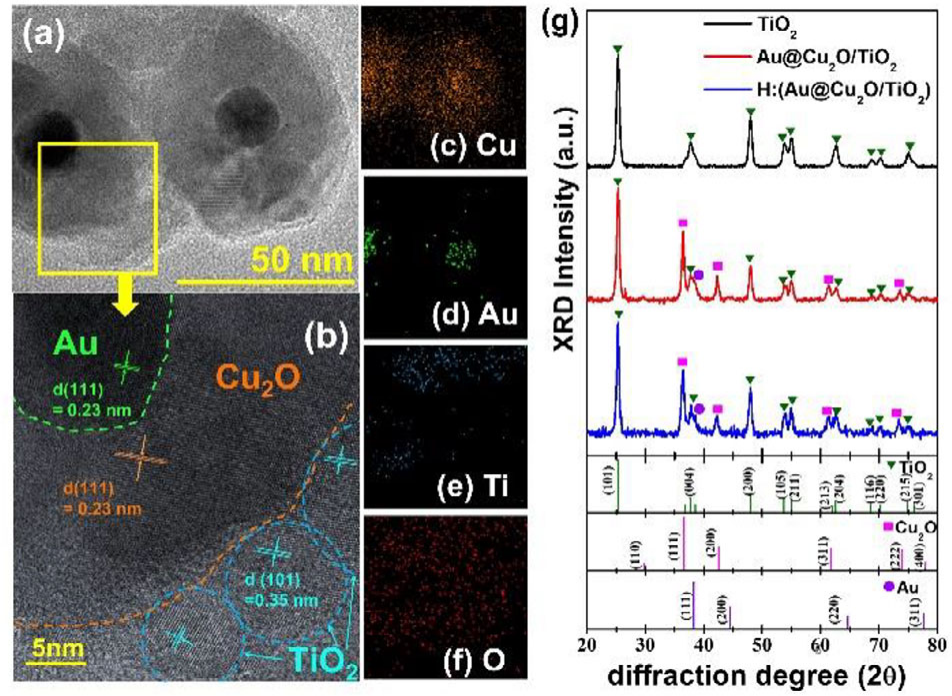
(a) TEM, b) HRTEM images of H:(Au@Cu_2_O/TiO_2_), TEM mapping analysis of c) Cu, d) Au, e) Ti, f) O, and g) X‐ray diffraction patterns of TiO_2_, Au@Cu_2_O/TiO_2,_ and H:(Au@Cu_2_O/TiO_2_) photocatalysts. JCPDS reference, anatanse TiO_2_: 21–1272; Cu_2_O:78‐2076, Au: 04–0784.

In the TEM image shown in Figure 1a, the nanostructure of the hydrogenated substrate, denoted as H:(Au@Cu_2_O/TiO_2_), is clearly observed. The Au nanoparticle (NP) is completely encapsulated by a Cu_2_O shell, forming a distinct core–shell architecture. Elemental mappings for Cu, Au, Ti, and O obtained by EDS are presented in Figure 1c–f. The Au signal is firmly confined within the Cu signal, confirming the core–shell configuration, while Ti signals are detected outside the Cu_2_O shell, indicating that TiO_2_ nanoparticles decorate the outer surface, as evident in Figure 1e. The HRTEM image in Figure 1b further reveals well‐defined lattice fringes corresponding to the (111) planes of Au and Cu_2_O, as well as the (101) planes of anatase TiO_2_. These results support the structural features of the composite: an Au core with a diameter of approximately 15 nm, a Cu_2_O shell of about 18 nm in thickness, and surface decoration with TiO_2_ nanoparticles roughly 13 nm in size. The measured d‐spacing values align well with previously reported data [12, 25], validating the successful synthesis of the H:(Au@Cu_2_O/TiO_2_) composite.

Based on the morphological analysis, the hierarchical core–shell–decorated structure of the composite is confirmed. Moreover, the XRD patterns of anatase TiO_2_ NPs, the Au@Cu_2_O/TiO_2_ composite, and the hydrogenated H:(Au@Cu_2_O/TiO_2_) sample are shown in Figure 1g. For comparison, standard JCPDS reference patterns for anatase TiO_2_ (No. 21–1272), Cu_2_O (No. 78–2076), and Au (No. 04–0784) are provided in the lower portion of Figure 1g. Diffraction peaks corresponding to TiO_2_ (▲), Cu_2_O (■), and Au (●) are clearly identified in both the composite and the hydrogenated sample, indicating high crystallinity. Importantly, no peaks corresponding to CuO are detected, suggesting that the Cu_2_O phase remains stable and intact following TiO_2_ decoration and hydrogenation treatment—highlighting the structural stability of the synthesized composite.

**FIGURE 2:**
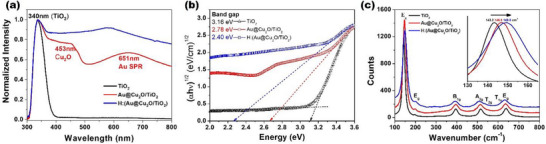
The a) UV–vis spectroscopy, b) Tauc plot, c) Raman spectroscopy of TiO_2_, Au@Cu_2_O/TiO_2_ and H:(Au@Cu_2_O/TiO_2_) photocatalysts.

The normalized UV–Vis absorption spectra (320–800 nm) of the samples are shown in Figure 2a, providing insight into their optical properties and photocatalytic performance. For the TiO_2_ NPs (black curve), a broad absorption peak centered at 340 nm—confined to the UV region—was observed, characteristic of anatase TiO_2_ [24]. To enhance visible light absorption, a core–shell Au@Cu_2_O structure was integrated with TiO_2_ NPs. Compared to pure TiO_2_, the Au@Cu_2_O/TiO_2_ composite (red curve) exhibited two additional pronounced absorption peaks at 460 and 620 nm. The peak at 460 nm corresponds to the Cu_2_O shell absorption [12, 26], while the broad band at 620 nm is attributed to the localized surface plasmon resonance (LSPR) of the Au core [27, 28, 29, 30]. For comparison, the UV–vis absorption spectra of Cu_2_O, Cu_2_O/TiO_2_ material are provided in the Figure S2a. A clear shoulder was observed at 470 nm, represented by the Cu_2_O absorption, further illustrating the separated absorption peaks information obtained in Au@Cu_2_O/TiO_2_ catalyst. Both Cu_2_O and Cu_2_O/TiO_2_ absorptions terminate at 615 nm, indicating the limitation of Cu_2_O application. Moreover, the Au‐assisted LSPR band shows a remarkable red‐shift relative to the typical 520 nm SPR peak of Au colloids in water, which is attributed to the high refractive index (n = 2.8–3.4) [31] of the surrounding Cu_2_O shell, in contrast to water's lower refractive index (n = 1.33) [28, 32]. For the hydrogenated composite, H:(Au@Cu_2_O/TiO_2_), a further enhancement in absorption was observed in the 450–800 nm range. This increase is associated with surface defect states introduced by hydrogenation, highlighting the effectiveness of the hydrogenation process [24, 33]. The extended absorption was also observed in the hydrogenated H:(Cu_2_O/TiO_2_) catalysts, confirming the hydrogenation effect on Cu_2_O clearly (see Figure S2a).

Figure 2b presents the corresponding Tauc plots used to estimate the optical bandgap (Eg) of each sample, based on the indirect transition nature of anatase TiO_2_ (n = 2). The intersection between the extrapolated tangent line (drawn from the linear region of the absorption edge) and the baseline of the absorption spectrum was used to determine the bandgap energies. The calculated bandgaps were 3.16 eV for TiO_2_, 2.78 eV for Au@Cu_2_O/TiO_2_, and 2.40 eV for H:(Au@Cu_2_O/TiO_2_). This progressive bandgap narrowing confirms the synergistic effect of the Au@Cu_2_O heterojunction and hydrogenation treatment, both of which significantly enhance the photocatalytic potential of the material.

The Raman spectra of TiO_2_, Au@Cu_2_O/TiO_2_, and H:(Au@Cu_2_O/TiO_2_), shown in Figure 2c, further support these observations. In the 100–800 cm^−^^1^ range, characteristic anatase TiO_2_ vibrational modes were observed at 143.2, 196.4, 396.0, 515.7, and 637.9 cm^−^^1^, corresponding to the Eg(1), Eg(2), B1g(1), A1g(1), and Eg(3) modes, respectively [34, 35]. Upon forming the core–shell Au@Cu_2_O structure decorated with TiO_2_ NPs, a new interfacial region between TiO_2_ and Cu_2_O was established. This interface introduces lattice strain and surface stress, resulting in a broadening and blue‐shifting of the TiO_2_ Eg peaks, as illustrated in the inset of Figure 2c. In the case of the hydrogenated composite, the main Eg mode further shifted to 149.6 cm^−^^1^, indicative of successful hydrogen incorporation.

Additionally, weak shoulder peaks at ∼536 and ∼615 cm^−^^1^ were detected in the spectra of Au@Cu_2_O/TiO_2_ and H:(Au@Cu_2_O/TiO_2_), which are assigned to the T_2g_ and T_1u_ vibrational modes of Cu_2_O, respectively [36, 37]. The infrared‐active T_1u_ mode was notably enhanced in the hydrogenated sample, suggesting increased oxygen vacancy concentration due to hydrogen treatment [38]. The oxygen vacancy intensity has been reasonably clarified from EPR spectroscopy as demonstrated in Figure 5. The enhanced generation of the oxygen vacancies in H:(Au@Cu_2_O/TiO_2_) with respect to Au@Cu_2_O/TiO_2_ was clearly by hydrogenation support this point. The enhancement of oxygen vacancies facilitates light‐induced charge separation under AM1.5G irradiation, reflecting the photoactive behavior well. No CuO‐related peaks were observed, confirming the preservation of the Cu_2_O phase and the absence of CuO formation, consistent with the structural analysis presented in Figure 1.

**FIGURE 3:**
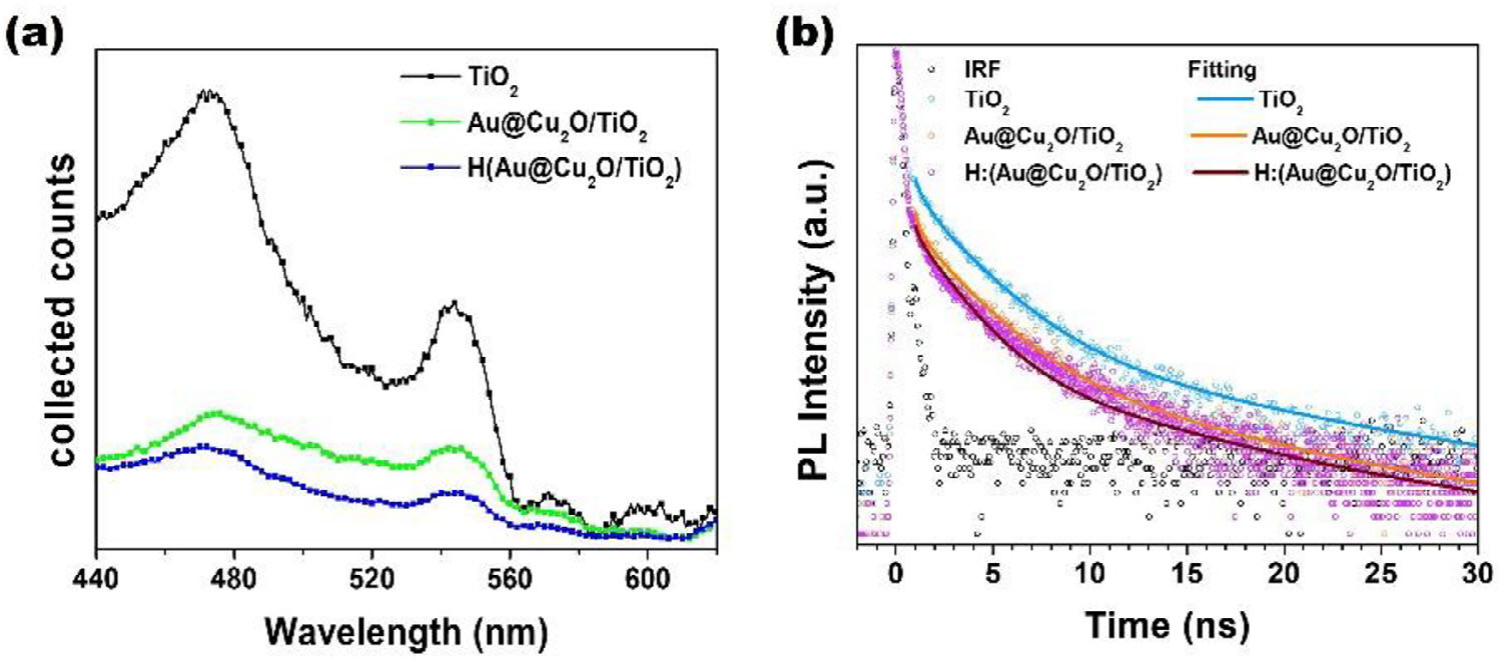
The a) steady state photoluminescence spectroscopy, b) TRPL spectroscopy of TiO_2_, Au@Cu_2_O/TiO_2,_ and H:(Au@Cu_2_O/TiO_2_) photocatalysts, λ_em_ 470 nm.

The steady‐state photoluminescence (PL) spectra of TiO_2_, Au@Cu_2_O/TiO_2_, and the hydrogenated counterpart H:(Au@Cu_2_O/TiO_2_) in the solution phase are shown in Figure 3a. The recombination of photoexcited electrons and holes would lead to photoemission [39]; TiO_2_ exhibits a strong emission peak at 470 nm, which is attributable to its inefficient separation of photogenerated electron–hole pairs and slow charge transfer dynamics. In contrast, both Au@Cu_2_O/TiO_2_ and H:(Au@Cu_2_O/TiO_2_) show a pronounced quenching of PL intensity, indicating the enhanced charge separation and efficient carrier transfer efficiency [40, 41]. This improvement is likely due to the formation of an effective *p–n* heterojunction between Cu_2_O and TiO_2_, which facilitates charge carrier separation [42, 43]. Meanwhile, a slight red shift had also noticed in hydrogenated composite H:(Au@Cu_2_O/TiO_2_) with respect to Au@Cu_2_O/TiO_2_. Due to the increase in defect states induced by the hydrogenation effect had been reported in hydrogenated nitride [33] and hydrogenated oxide semiconductors [44] with an observation of PL shifted toward lower energy as a result. Furthermore, the band gap decreasing phenomenon was observed with H:(Au@Cu_2_O/TiO_2_) in the Tauc plot, which also supports this result as shown in Figure 2b. To further investigate the charge transfer kinetics and interfacial dynamics, time‐resolved photoluminescence (TRPL) measurements were conducted for TiO_2_, Au@Cu_2_O/TiO_2_, and H:(Au@Cu_2_O/TiO_2_) at an emission wavelength of 470 nm, as shown in Figure 3b. All TRPL measurements were performed using 320 nm excitation, with emission collected at the band‐edge of TiO_2_ to directly probe the decay behavior of photogenerated carriers in TiO_2_. The decay curves were fitted using a tri‐exponential function:

(1) $$I\left(t\right)=I_{0}+A_{1}\;e^{-t/\tau_{1}}+A_{2}\;e^{-t/\tau_{2}}+A_{3}\;e^{-t/\tau_{3}}$$

For the pristine TiO_2_, its TRPL decay curve was deconvoluted to 3 components representing the process of the surface‐related radiative and non‐radiative recombination for TiO_2_ with τ_3_ = 0.5 ns and τ_2_ = 2.58 ns, respectively. The trace longer lifetime in τ_1_ ∼ 12 ns was attributed to the recombination of free excitons, as has been elucidated in a similar system [29]. The recombination of photoexcited electrons and holes would lead to photoemission [39], which can be estimated from the average carrier lifetime (τ_av_) according to the following equation [30]:

(2) $$\tau_{av}=\left(A_{1}\tau_{1}^{2}+A_{2}\tau_{2}^{2}+A_{3}\tau_{3}^{2}\right)/\;\left(A_{1}\tau_{1}+A_{2}\tau_{2}+A_{3}\tau_{3}\right)$$

The decreased intensity observed in steady state PL of Au@Cu_2_O/TiO_2_ was attributed to the fast electron transfer process from TiO_2_ to Cu_2_O according to the Z‐scheme configuration, and a clear reduction in lifetime was observed across the series—from 4.24 ns for TiO_2_ to 3.14 ns for Au@Cu_2_O/TiO_2_. The defect states created by hydrogenation would further reduce the lifetime to 2.96 ns for H:(Au@Cu_2_O/TiO_2_)—demonstrating accelerated charge transfer processes in the modified systems [25, 42] which also offer a deeper insight into the dynamic charge transfer mechanisms. Based on the reciprocal lifetime difference, the effective interfacial charge‐transfer rates (*k_et_*) was estimated using

(3) $$k_{et}=\frac{1}{\tau_{av,composite}}\;-\frac{1}{\tau_{av,TiO_{2}}}$$

Accordingly, the values of *k*_et_ were determined to be 8.21 × 10^7^ s^−1^ for Au@Cu_2_O/TiO_2_ and 1.02 × 10^8^ s^−1^ for H:(Au@Cu_2_O/TiO_2_), respectively. The higher transfer rate in the hydrogen‐treated sample suggests a more efficient carrier extraction from Cu_2_O to TiO_2_, consistent with its enhanced photocatalytic hydrogen evolution performance.

**FIGURE 4:**
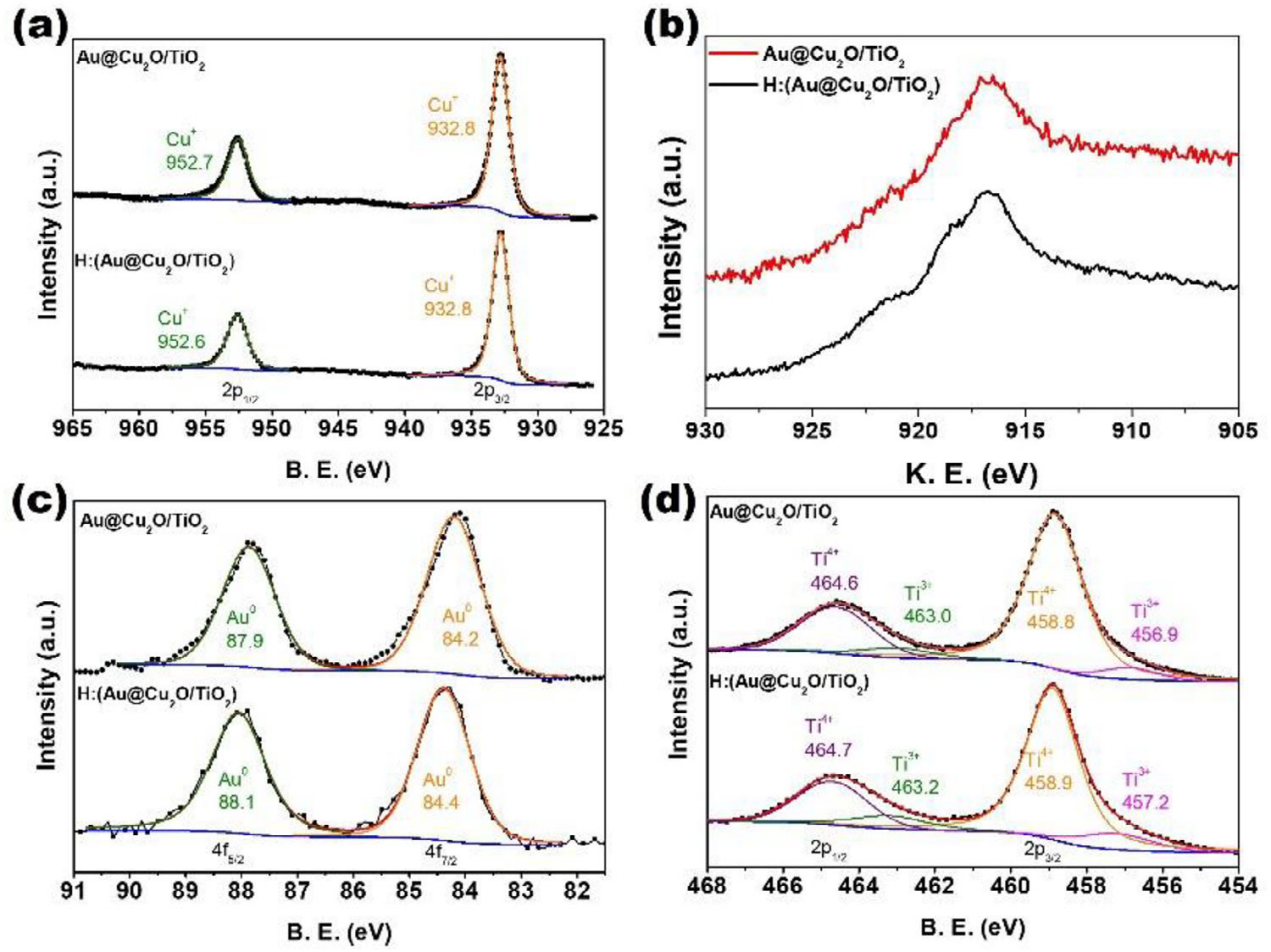
XPS spectroscopy of a) Cu 2p, b) Cu LMM, c) Au 4f and d) Ti 2p for Au@Cu_2_O/TiO_2_ and H:(Au@Cu_2_O/TiO_2_) photocatalysts.

The XPS spectra illustrating the chemical states of Au@Cu_2_O/TiO_2_ and H:(Au@Cu_2_O/TiO_2_), based on surface analysis, are presented in Figure 4a–d. For the Cu binding energy analysis, two distinct peaks were identified at 932.8 and 952.7 eV for Au@Cu_2_O/TiO_2_, and at 932.8 and 952.6 eV for H:(Au@Cu_2_O/TiO_2_). These peaks correspond to the Cu 2p_3/2_ and 2p_1/2_ signals of Cu^+^ in Cu_2_O, consistent with previously reported studies [31, 42, 45]. No peaks corresponding to Cu^2+^ were detected, indicating the presence of only the Cu_2_O lattice. Although the copper metal plays a significant role in photo‐illumination processes, the binding energies of Cu^0^ and Cu^+^ are too close to be clearly distinguished in Figure 4a. Figure 4b shows the Cu LMM Auger electron spectra for Au@Cu_2_O/TiO_2_ (red line) and H:(Au@Cu_2_O/TiO_2_) (black line). The Cu kinetic energy (KE) was calculated using the formula KE = 1486.6 eV‐BE, where 1486.6 eV corresponds to the Al Kα X‐ray energy. In this spectrum, a distinct Cu^+^ peak at 916.9 eV was observed for both samples, with no clear indication of metallic Cu, suggesting that the Cu_2_O crystal structure remains well preserved [46].

As shown in Figure 4b, the Au peaks exhibit sharp doublets at 84.2 and 87.9 eV for Au@Cu_2_O/TiO_2_, and at 84.4 and 88.1 eV for H:(Au@Cu_2_O/TiO_2_), corresponding to the Au^0^ 4f_7/2_ and 4f_5/2_ binding energies, respectively [27, 28, 29]. This indicates that the Au core is well preserved within the Cu_2_O shell in both composites. In Figure S3, pristine TiO_2_ displays two prominent peaks at 458.5 and 464.3 eV, corresponding to the stable Ti^4+^ 2p_3/2_ and 2p_1/2_ states of the TiO_2_ lattice, with a spin–orbit splitting of 5.7 eV, consistent with literature reports [24, 26]. Similarly, Figure 4d shows well‐defined Ti^4+^ peaks at 458.8 and 464.6 eV for Au@Cu_2_O/TiO_2_, and at 458.9 and 464.7 eV for H:(Au@Cu_2_O/TiO_2_) [29, 42]. Additionally, a small shoulder preceding the Ti^4+^ peaks was observed, which, upon deconvolution, corresponds to Ti^3+^ 2p_3/2_ and 2p_1/2_ peaks at 456.9 and 463.0 eV for Au@Cu_2_O/TiO_2_, and at 457.2 and 463.2 eV for H:(Au@Cu_2_O/TiO_2_), respectively [42, 47]. The asymmetric Ti^3^^+^ peak arises from the interaction between Au@Cu_2_O and TiO_2_. The integrated Ti^3+^ peak areas were compared, revealing that the Ti^3+^ content increased from approximately 5.8% in Au@Cu_2_O/TiO_2_ to 10.5% in the hydrogenated sample (H:(Au@Cu_2_O/TiO_2_)). Controlling the Ti^3+^ concentration is critical for enhancing photocatalytic performance, as hydrogenation induces the formation of additional surface oxygen vacancies, which is reflected in the increased Ti^3+^ content [42]. Table S3 summarizes the Ti^3+^ and Ti^4+^ percentages for all photocatalysts investigated in this study. The XPS of the H:(Au@Cu_2_O/TiO_2_) substrate before and after irradiation were presented and compared in Figure S6; the binding energy band has a well‐retained and no clear additional peak is noted after long‐time illumination, indicating a well‐preserved structure for the composite catalyst fabricated in this work.

**FIGURE 5:**
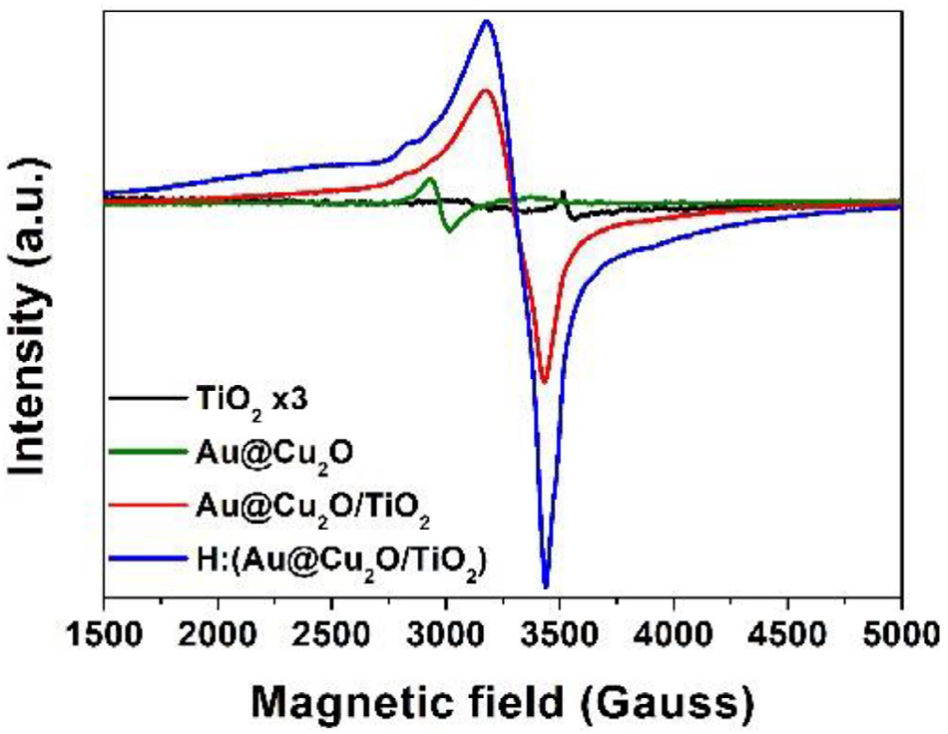
The EPR spectra of TiO_2_, Au@Cu_2_O, Au@Cu_2_O/TiO_2_ and H:(Au@Cu_2_O/TiO_2_) sample.

By comparing the EPR spectra obtained from TiO_2_, Au@Cu_2_O, Au@Cu_2_O/TiO_2_, and H:(Au@Cu_2_O/TiO_2_) powders, as demonstrated in Figure 5, the low intensity of unpaired electrons was observed in TiO_2_ and Au@Cu_2_O. The EPR intensity was extensively increased in Au@Cu_2_O/TiO_2_, clearly demonstrating the trapped electrons induced by the oxygen vacancies, and the chemical bond formed in Au@Cu_2_O‐TiO_2_ was expected with the interaction of spin–orbit coupling observed between TiO_2_ and Au@Cu_2_O [48]. The intensity of unpaired electrons was further strengthened in H:(Au@Cu_2_O/TiO_2_), reflecting the effect of hydrogenation effect.

To optimize the efficiency of hydrogen photocatalytic evolution, Au@Cu_2_O/TiO_2_ composites were prepared with varying mass ratios of Au@Cu_2_O to TiO_2_ (1:10, 1:5, 1:2, and 1:1) by controlling the weights of the corresponding nanoparticles during the blending process. After undergoing the hydrogenation treatment in methanol vapor, as previously described, the hydrogen evolution rates of the hydrogenated substrates under 5 h of AM 1.5 solar illumination were evaluated, as shown in Figure S4a, with average values presented as bar graphs (in mmol g^−1^ h^−1^). Meanwhile, the reactivity toward the exposed active sites was controlled by determining the Specific Surface Area (SSA) from N_2_ adsorption–desorption isotherms at 77 K using a Micromeritics ASAP 2020 analyzer, applying the Brunauer–Emmett–Teller (BET) and the Barrett–Joyner–Halenda (BJH) model for analysis. Samples were degassed at 120°C before measurement. The specific surface area acquired from 50 mg reacting powders with SSA 60.9, 65.7, 68.2, and 68.2 m^2^ g^−1^ for 1:1, 1:2, 1:5, and 1:10 catalysts, respectively, as presented in Figure S4a. The photocatalytic HER results follow the trend of SSA in 1:1 and 1:2 catalysts, indicating the dependence on reactivity to the exposed active site [49]. A quantitative comparison of the BET specific surface areas and the corresponding HER activities, including mass‐ and surface‐area‐normalized values for different Au@Cu_2_O/TiO_2_ ratios, is summarized in Table S4. However, the observed positive HER trend ceased in 1:5 substrate and decreased dramatically to 2.39 mmol g^−1^ h^−1^ in the 1:10 catalysts with a slightly higher SSA due to the insufficient Au@Cu_2_O content. The highest HER was observed at the 1:2 ratio. The actual element ratio was evaluated from ICP‐MS analyses, which gave the at% of the H:(Au@Cu_2_O/TiO_2_) sample with Ti 39.7%, Cu 24.6%, and Au 3.9% was provided. The Au@Cu_2_O: TiO_2_ ratio was computed to be 31:69 (mol %) for our optimized sample. To further evaluate the photocatalytic performance, substrates with different catalyst loadings (2, 5, and 10 mg) were dispersed in 40 mL of 20% (v/v) methanol solution prior to illumination. As illustrated in Figure S4b, the 2 mg catalyst produced an HER of 8.7 mmol g^−1^ h^−1^, while the 5 mg sample exhibited the highest HER of 9.3 mmol g^−1^ h^−1^. Interestingly, the 10 mg catalyst showed a significant drop in HER, nearly halving the performance due to severe nanoparticle aggregation.

**FIGURE 6:**
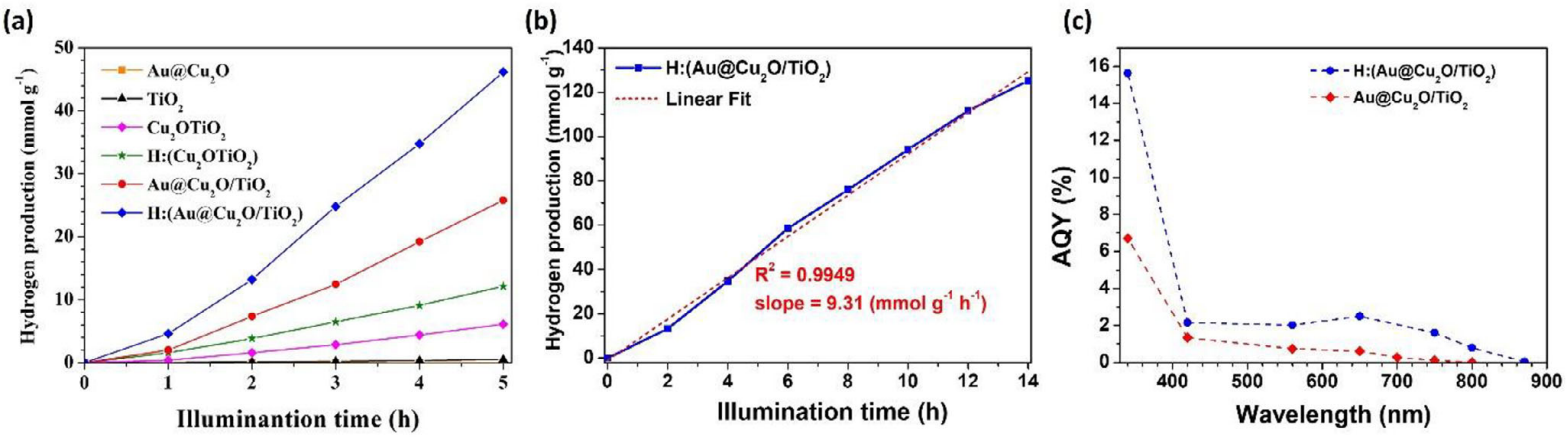
Hydrogen production activity comparison of 5 mg photocatalyst powders mixed with 20% v/v methanol sacrificial solution under AM1.5 illumination. a) Hydrogen production results of related substrates obtained in 5 h, b) HER reliability measurement of H:(Au@Cu_2_O/TiO_2_) in extended hours, c) AQY measurement under single wavelength illumination in the UV–Vis–NIR range.

To provide a comprehensive overview, Figure 6a presents the full photocatalytic response under 5 h of AM 1.5 illumination for anatase TiO_2_, Au@Cu_2_O core–shell structures, the Au@Cu_2_O/TiO_2_ composite, and the hydrogenated version (H:Au@Cu_2_O/TiO_2_). Based on the optimization results, 5 mg of photocatalyst with the 1:2 Au@Cu_2_O/TiO_2_ ratio was used in 40 mL of 20% methanol solution for further testing. To determine the role of methanol in the redox reaction, a comparative experiment was carried out with pure water. As demonstrated in Figure S7, the (H:Au@Cu_2_O/TiO_2_) sample was almost inactive in pure water (HER = 0.0004 mmol g^−1^ h^−1^); the efficient methanol scavenger was conducted by the giant discrepancy in‐between with 20% methanol profound photocatalytic HER result (9.3 mmol g^−1^ h^−1^) discussed above. The limited UV absorption characteristics of TiO_2_ lead to its poor HER result (0.103 mmol g^−1^ h^−1^). The averaged HER of the Cu_2_O/TiO_2_ catalysts, 1.26 mmol g^−1^ h^−1^, suggests that the recombination of photon‐excited electron and hole pair was effectively reduced by the interfacial *p–n* heterojunction formed between *p‐*type Cu_2_O and *n*‐type TiO_2_ [19]. For the hydrogenated material H:(Cu_2_O/TiO_2_), the HER was doubled to 2.40 mmol g^−1^ h^−1^ due to oxygen vacancies induced by the hydrogenation effect, which was clearly confirmed in this system. The introduction of direct electron injection from the plasmonic Au, the Au@Cu_2_O/TiO_2_ composite, exhibited a significantly enhanced HER of 5.51 mmol g^−1^ h^−1^, remarkably. Following hydrogenation, the HER of H:(Au@Cu_2_O/TiO_2_) further increased to 9.3 mmol g^−1^ h^−1^ due to the oxygen vacancies induced by the hydrogenation effect, which enhanced charge carrier mobility. To explore the hydrogenation effect on an isolated system, the controlled HER experiments with Au@Cu_2_O, TiO_2_, hydrogenated H:(Au@Cu_2_O), and H:TiO_2_ material had provided by bar graphs presented in Figure S4c. The HER result of Au@Cu_2_O was measured to be 0.003 mmol g^−1^ h^−1^, attributable to the fast e‐h pair recombination within the Cu_2_O shell. Surface defects may be created by the hydrogenation effect to trap an excited electron; the HER of H:(Au@Cu_2_O) was slightly enhanced to 0.068 mmol g^−1^ h^−1^ under the consecutive 5 h AM 1.5G illumination, supporting this point. The photoactivity of the coreshell system is thus seen to be greatly limited without TiO_2_ for a more effective e‐h separation. TiO_2_ material is limited to UV absorption due to its large bandgap, an extended absorption to the Vis range with hydrogenated samples has been demonstrated in our earlier publication [23] for the oxygen vacancy concentration enhancement by the increasing ‐OH from hydrogenated TiO_2_. Herein, the HER performance improvement to 0.262 mmol g^−1^ h^−1^ was observed with respect to the TiO_2_ of HER 0.103 mmol g^−1^ h^−1^ as discussed above. Finally, when compared to the isolated Au@Cu_2_O structure, this corresponds to an astonishing enhancement of approximately 1836 and 3096 fold for the Au@Cu_2_O/TiO_2_ and H:(Au@Cu_2_O/TiO_2_) composites, as shown in Figure 6a, respectively. Prolonged AM 1.5 illumination for up to 14 h confirmed the stability and reliability of the hydrogenated photocatalyst, with sustained HER performance (Figure 6b). The delicate designed H:(Au@Cu_2_O/TiO_2_) was provided with a comprehensive discussion on electron transfer.

To further investigate the photocatalytic behavior under visible light, hydrogen production was measured under monochromatic light using band‐pass filters. The AQY values of Au@Cu_2_O/TiO_2_ and H:(Au@Cu_2_O/TiO_2_) across the UV–Vis–NIR range are shown in Figure 6c. The pure Au@Cu_2_O structure exhibited poor HER due to inefficient electron‐hole separation. However, the formation of a *p–n* junction in the Au@Cu_2_O/TiO_2_ composite significantly improved charge separation, extending the AQY up to 650 nm with a value of 0.6%. The hydrogenated composite displayed exceptional performance, achieving an AQY of 15.6% in the UV region, and 2.2%, 2.0%, 2.5% and 1.6% at 420, 560, 650, and 750 nm, respectively, in the visible region. Furthermore, the AQY extended into the NIR region, reaching 0.79% and 0.05% at 800 and 890 nm. Finally, the visible‐light‐driven photocatalytic performance of the H:(Au@Cu_2_O/TiO_2_) composite was compared with previously reported Au–TiO_2_ and Cu_2_O–TiO_2_ systems under solar illumination, as summarized in Table S2. The HER and AQY data clearly highlight the promising potential of this novel hydrogenated Au@Cu_2_O/TiO_2_ composite for efficient photocatalytic hydrogen evolution.

**FIGURE 7:**
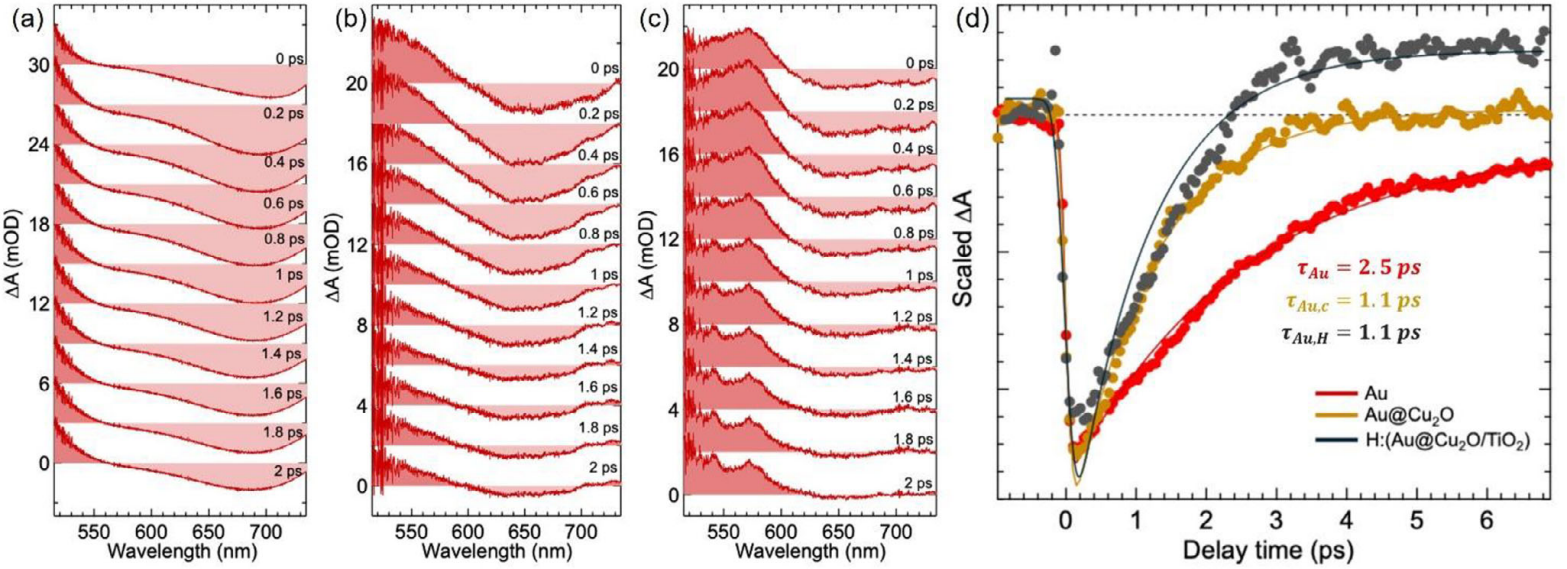
Femtosecond transient absorption spectra of a) Au, b) Au@Cu_2_O, and c) H:(Au@Cu_2_O/TiO_2_) film sample. The combined transient kinetic profiles representing the thermalization process of d) Au, Au@Cu_2_O, and H:(Au@Cu_2_O/TiO_2_) with τ_*Au*_, τ_*Au*, *c*_ τ_*Au*, *H*_ listed for comparison. The nanoparticle films were prepared by the drop‐casting method. The pump wavelength was set at 397 nm.

The Femtosecond transient absorption spectral (TAS) studies were performed on pristine Au, Au@Cu_2_O, and H:(Au@Cu_2_O/TiO_2_) heterojunction nanoparticle films to investigate the plasmonic charge separation processes between multiple Au/Cu_2_O and Cu_2_O/TiO_2_ interfaces. The samples were excited using a 397 nm femtosecond laser pulse, and the absorption changes in plasmonic gold nanoparticles were monitored using a probe pulse generated from a white‐light continuum. Bare TiO_2_ and Cu_2_O nanoparticles could not produce any TA signals under the current excitation fluence, suggesting weaker absorption cross‐sections at 400 nm for these samples. The TAS profiles of Au nanoparticles show a depletion of the plasmonic band of Au immediately after photoexcitation, with band minima at 690 nm as presented in Figure 7. The depleted plasmonic bands showed recovery with increasing delay time between pump and probe pulses. The recombination dynamics of Au nanoparticles exhibit a recovery time (τ_*Au*_) of 2.5 ps, accompanied by a small offset signal attributed to a long‐lived decaying component. The 2.5 ps decaying component was assigned to relaxation due to the thermalization process involving electron–phonon (e‐ph) interactions [49, 50, 51]. Plasmon damping occurs within 10 fs following photoexcitation, resulting in a non‐thermal distribution of electrons and holes that undergo thermalization with the lattice via electron–phonon (e‐ph) interactions [52].

The Au@Cu_2_O and H:(Au@Cu_2_O/TiO_2_) nanoparticles also show depletion of Au plasmonic bands following photoexcitation. However, the thermalization process becomes faster due to charge separation from plasmonic Au nanoparticles to the Cu_2_O. The decay lifetime of the plasmonic band of Au in coreshell Au@Cu_2_O (τ_*Au*, *c*_) and hydrogenated H:(Au@Cu_2_O/TiO_2_) (τ_*Au*,*H*_) sample at 650 nm was estimated to be 1.1 ps as demonstrated in Figure 7. Earlier reports on Au/semiconductor nanoparticles showed a similar reduction in thermalization times due to the reduced population of hot electrons, resulting from the rapid injection of hot electrons into the semiconductors, which is in agreement with the observed results here [51, 53, 54]. Typically, the quantum yield (QY) of hot electron transfer from metal to semiconductor is usually modelled using Fowler's equation, given by [55]

(4) $$\mathrm{QY}\left(\omega \right)={\left(\hbar \omega -E_{b}\right)}^{2}\;\left(4E_{F}\hbar \omega \right)$$

where *E_F_* represents Fermi level of the metal, *E_b_* represents the Schottky barrier height between the metal and semiconductor, and ℏω represents the excitation energy. For the 397 nm excitation, the QY of hot electron transfer was estimated to be close to 7.8%. The Fermi level of gold used in the calculation is 4.03 eV and the barrier height is estimated to be 1.14 eV based on the difference between Cu 3d E_CBM_ and the Fermi level.

The hot electron transfer process between Au and Cu_2_O can be further understood from the band energy diagram obtained from the UPS experiments shown in Figure S10. Au nanoparticles show a work function at −4.03. The Fermi levels of Cu_2_O and TiO_2_ before contact were located at −3.5 and −4.2 eV. As a result of band bending at the interface, a Z‐scheme heterojunction was formed between TiO_2_ and Cu_2_O by compensating the electron‐hole recombination, leaving excess electrons on Cu_2_O and holes on TiO_2_ for promoted reduction and oxidation reactions, separately.

**FIGURE 8:**
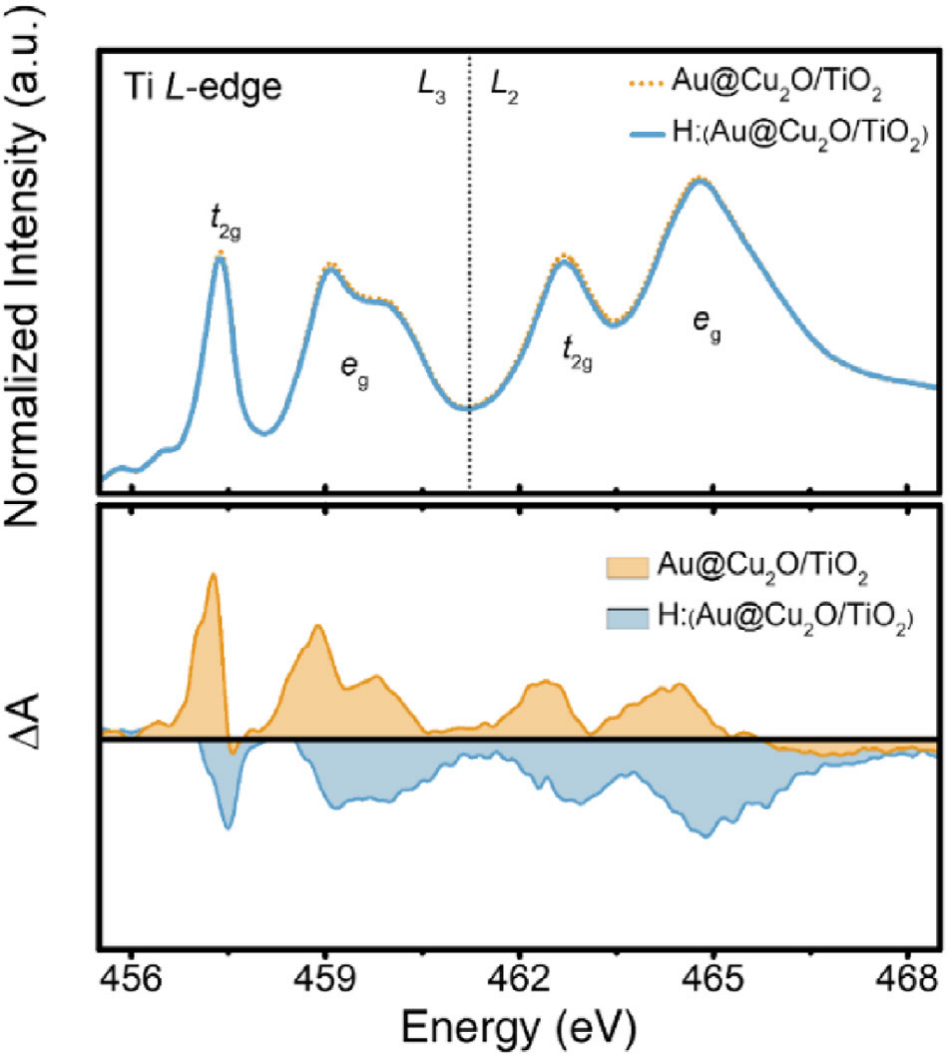
Normalized Ti *L*‐edge XANES spectrum of Au@Cu_2_O/TiO_2_ and H:(Au@Cu_2_O/TiO_2_) under illumination. The colour‐filled areas represent the intensity differences between illumination (AM1.5) and darkness states (Δ*A* = *A*_illumination_ – *A*_darkness_).

Although these composite photocatalysts have been extensively characterized using multiple conventional techniques, the fundamental electronic and structural properties of their composite elements warrant further investigation via synchrotron radiation‐based methodologies. To this end, in situ soft XAS was employed to probe illumination‐induced variations in the charge transfer states from Au@Cu_2_O/TiO_2_ and subsequent hydrogenation treatments. As illustrated in Figure 8, two prominent features—*t*_2g_ and *e*_g_—appear in the Ti L‐edge XAS spectra, corresponding to electron transitions from the 2p*_3/2_* core level to unoccupied 3d orbitals above the Fermi level. Based on these spectral results, the Ti valence state remains largely stable; however, a slight alteration in the *t*_2g_/*e*_g_ intensity ratio suggests subtle changes in the density of unoccupied states following hydrogenation.

Furthermore, the differential spectral intensities of Au@Cu_2_O/TiO_2_ and hydrogenated H:(Au@Cu_2_O/TiO_2_) under solar irradiation are represented by two color‐filled regions (∆*A*) in Figure 8. The positive ∆*A* values (∆*A* > 0) observed for Au@Cu_2_O/TiO_2_ are attributed to a limited generation of photoexcited electrons from the Au@Cu_2_O component, thereby indirectly promoting electron‐hole recombination. In contrast, the comparatively negative ∆*A* values for H:(Au@Cu_2_O/TiO_2_) suggest that hydrogenation facilitates more efficient photocarrier separation and transfer, particularly across the Au@Cu_2_O/TiO_2_ heterojunction interface. This modification enhances photoelectron mobility toward the TiO_2_ domain, resulting in the rapid separation of hot carriers and a pronounced suppression of recombination processes, as evidenced by the in situ soft XAS results.

The chopped PEC photocurrent curves (with 50s ON‐OFF cycles) are presented in Figure S8a, the TiO_2_ sample gave a stable photocurrent of 10.7 µA cm^−2^ with a fast photo‐response time under AM1.5 illumination without applied bias. A reverse (negative) photocurrent was observed in the Au@Cu_2_O electrode, which further shifts to a higher negative photocurrent under light, corresponding to hole transfer to the indium tinoxide (ITO) glass due to the *p‐*type Cu_2_O shell. However, its composite with TiO_2_, i.e., Au@Cu_2_O/TiO_2_ (with relatively higher TiO_2_ concentration) showed photocurrent up to 23.6 µA cm^−2^, while after hydrogenation, it increased to 30.3 µA cm^−2^ for the optimized H:(Au@Cu_2_O/TiO_2_) (1:2) sample as demonstrated in Figure S8a. After hydrogenation, the additional defect levels evolved below CB helped to decrease the electron‐hole recombination, resulting in an improved photocatalytic performance [56]. These additional trap levels near the CB of TiO_2_ can also serve as the smooth steps for the coupling of the TiO_2_ conduction band and the Cu_2_O valence band in the Z‐scheme. Interestingly, the elongated decay of photocurrent under dark as observed in Au@Cu_2_O/TiO_2_ transformed to a sharp decay after its hydrogenation (in H:(Au@Cu_2_O/TiO_2_)), indicating the removal of some intermediate trap state that was adversely affected the photogenerated electron transfer for water splitting. Since the CB of Cu_2_O plays a major role in electron transfer for H_2_ evolution, a higher concentration of TiO_2_ (Au@Cu_2_O: TiO_2_; 1:10 ratio) might block light absorption by the Cu_2_O active surface, which subsequently resulted in a lower photocurrent density as presented in Figure S8b. Moreover, during photocurrent measurements, it has been observed that except TiO_2_, all other heterojunction photocatalysts need to be activated in a 3‐electrode system atleast once under AM1.5G light irradiation to stabilize the photocurrent cycles under ON–OFF conditions. After electrode activation, all these samples showed the same photocurrent trend throughout the PEC measurement without any performance degradation (Figure S8), which revealed the stability of the prepared heterojunction photocatalysts in the methanol/water system under AM1.5G light.

### 2.1 Proposed Mechanism

**SCHEME 1:**
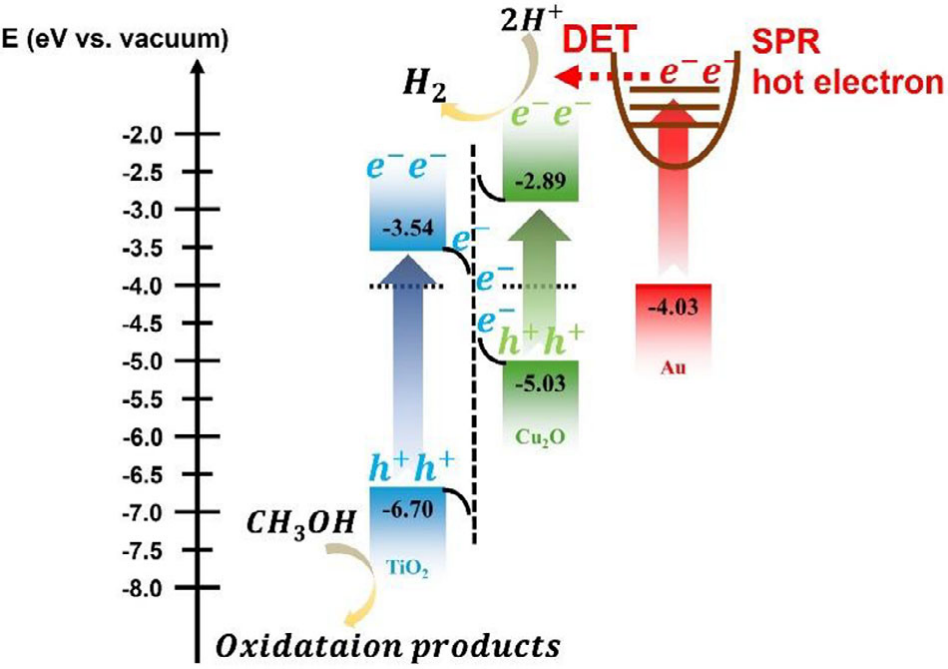
Proposed band alignment of H:(Au@Cu_2_O/TiO_2_) after contact.

To gain insight into the band structure and interfacial charge transfer mechanism within H:(Au@Cu_2_O/TiO_2_), ultraviolet photoelectron spectroscopy (UPS) was conducted to determine the energy distribution of valence band electrons. From the UPS spectra (Figure S5), key parameters such as the Fermi level and valence band maximum (E_VBM_) were extracted. The UPS measurements employed a He‐I light source with a photon energy of 21.2 eV. High‐energy and low‐energy cutoff values were used to calculate the E_VBM_ values relative to vacuum: Cu_2_O at −5.03 eV and TiO_2_ at −6.70 eV. By integrating these valence band positions with the bandgap energies (E_B_) obtained from Tauc plots (E_VBM_ = E_B_ + E_CBM_) [57]; Figure 2b), the full band structure of the H:(Au@Cu_2_O/TiO_2_) composite was constructed with Fermi level alignment (Figure S10). A schematic of the proposed carrier relaxation mechanism of the heterojunction is shown in Scheme 1. Following photoexcitation, hot electrons are generated in the plasmonic Au nanoparticles in addition to the photogenerated electrons in the Cu_2_O and TiO_2,_ as depicted in Scheme 1. The highly energetic hot electrons, which are rapidly injected into the conduction band of Cu_2_O via a direct electron transfer (DET) process upon light irradiation had determined by the TAS measurement. The population of remnant free carriers in the heterojunction samples can be visualized in the form a long‐lived, broader photoinduced absorption bands in the TA spectra of heterojunction samples in comparison to pristine Au nanoparticles. The Cu_2_O/TiO_2_ *p–n* junction effectively separates the photogenerated charge carriers, enabling efficient electron migration resulting in a remarkable hydrogen evolution enhancement from 2.4 mmol g^−1^ h^−1^ to 9.3 mmol g^−1^ h^−1^ for H:(Cu_2_O/TiO_2_) and H:(Au@Cu_2_O/TiO_2_), respectively. This mechanism is further supported by Ti L‐edge XAS analysis, which indicates that the conduction band of TiO_2_ receives a greater number of hot electrons under illumination. The TiO_2_ electrons were transferred to the VB of Cu_2_O, where TiO_2_ electrons recombine with Cu_2_O holes, leading to the accumulation of electrons of Cu_2_O site with HER enhancement upon the Z‐scheme structure discussed above, thereby confirming the proposed charge transfer pathway.

(5) $$Cu_{2}O\to Cu_{2}O+e^{-}+h^{+}$$

(6) $$H^{+}+e^{-}\to \frac{1}{2}H_{2}$$

Additionally, due to the lower valence band position of TiO_2_ (−6.7 eV) relative to that of Cu_2_O (−5.03 eV), photoinduced holes in TiO_2_ can readily migrate to the TiO_2_ surface. To complete the reaction cycle, methanol serves as an effective hole scavenger, being oxidized by photoinduced holes at the TiO_2_ surface, thereby further enhancing the separation of photogenerated charge carriers [19, 56, 58] for overall photocatalytic efficiency improvement. Among all, the hydrogen production rate of H:(Au@Cu_2_O/TiO_2_) composite exhibited dramatic improvements—approximately 3096 fold compared to pure Au@Cu_2_O and 81 fold relative to pure TiO_2_ as shown in Figure 6a.

(7) $$TiO_{2}\to TiO_{2}+h^{+}+e^{-}$$

(8) $$OH^{-}+h^{+}\to OH\cdot$$

(9) $$CH_{3}OH+OH\cdot \to oxidation\;products$$

## 3 Conclusion

We have developed precisely engineered heterostructure composites—Au@Cu_2_O/TiO_2_ and its hydrogenated counterpart H:(Au@Cu_2_O/TiO_2_)—based on an Au@Cu_2_O core–shell architecture further decorated with anatase TiO_2_ nanoparticles, exhibiting excellent performance in photocatalytic hydrogen evolution. The Au@Cu_2_O core–shell structure facilitates strong visible light absorption, where energetic hot electrons generated via the LSPR of Au are effectively injected into the Cu_2_O shell. The decay lifetime of the plasmonic Au band was reduced to 1.1 ps for Au@Cu_2_O and H:(Au@Cu_2_O/TiO_2_) with respect to 2.5 ps for the pristine Au had provided insight of effective interface transfer from TAS results analyses. Resulting from the Z‐scheme mechanism, the formation of a *p–n* heterojunction between Cu_2_O and TiO_2_ significantly promotes charge separation and accelerates electron transfer to Cu_2_O, leading to a substantial enhancement in hydrogen production. Notably, an AQY of 2.5% at 650 nm was achieved. To elucidate the pathway of photo‐induced charge carrier transfer, a comprehensive band alignment and reaction mechanism have been proposed, supported by detailed UPS, TAS, and XAS analyses.

To optimize photocatalytic hydrogen production, the Au@Cu_2_O to TiO_2_ mass ratio was varied from 1:10 to 1:1. The highest hydrogen evolution rate achieved was 9.3 mmol g^−^^1^ h^−^^1^ under AM 1.5 simulated sunlight. This rate represents a remarkable case of 3096‐fold and 81‐fold enhancements compared to pure Au@Cu_2_O and anatase TiO_2_ nanoparticles, respectively. In exploring the application of renewable energy, the synergistic effects of the composites were thoroughly analyzed, by integrating the direct injection of plasmonic hot electrons and the dedicate spatial separated redox system, the remarkable photocatalytic performance of the heterostructured composite was provided in this work.

## 4 Experimental Section

### 4.1 Chemicals

All chemicals were stored under dry conditions and used without further purification. Deionized (DI) water (resistivity > 18.2 MΩ·cm at 25°C) was employed throughout the experiments. The following reagents (with chemical names, purity grades, and supplier details) were used: Titanium dioxide (TiO_2_, 99.7%, Aldrich), Sodium hydroxide (NaOH, 97%, UniRegion Bio‐Tech), L(+) Ascorbic acid (C_6_H_8_O_6_, 99%, ACROS), Copper sulfate pentahydrate (CuSO_4_·5H_2_O, 99%, Riedel‐de Haën), Sodium citrate dihydrate (C_6_H_5_Na_3_O_7_·2H_2_O, MACRON), Hydrogen tetrachloroaurate(III) trihydrate sodium (HAuCl_4_·3H_2_O, 99.99%, Thermo Scientific).

### 4.2 Synthesis of Au@Cu_2_O/TiO_2_ nanoparticles

The experimental methodology is illustrated in Figure S1. In summary, core–shell Au@Cu_2_O nanoparticles were synthesized via a rapid deposition of Cu_2_O onto pre‐formed Au colloids. Initially, the Au colloids were prepared following the well‐established Turkevich method for the reduction of HAuCl_4_ [59]. Specifically, 2.5 mL of 0.01 m HAuCl_4_ and 0.2 mL of 0.5 m sodium citrate were each added separately to 97.5 mL of DI water under vigorous boiling in a two‐neck flask. The yellow Au^+^ ions were rapidly reduced to red Au^0^ nanoparticles [59, 60]. The reaction mixture was maintained at reflux for an additional 10 min with a condenser attached to ensure complete reduction, after which the red Au colloidal solution was allowed to cool naturally to room temperature without agitation.

Subsequently, two precursor solutions—designated as Solution I and Solution II—were prepared for the formation of the Au@Cu_2_O core–shell structures. Solution I comprised of 4 mL of 0.01 m CuSO_4_ mixed with 26 mL of DI water. Solution II consisted of 3 mL 1 m NaOH and 1 mL 0.1 m L‐ascorbic acid (LAA), dissolved in 43 mL of DI water. As depicted in Figure S1, the synthesis of Au@Cu_2_O was initiated by sequentially introducing the Au colloid solution and the NaOH/LAA mixture into the CuSO_4_ solution under continuous stirring at 35°C.

The immediate appearance of a greenish hue signified the onset of Cu_2_O formation through chemical reduction [31], indicating the successful generation of Au@Cu_2_O nanoparticles. After stirring for an additional 10 min to ensure complete reaction, the resulting green Au@Cu_2_O nanoparticles were collected by repeated centrifugation and washing. The purified powders were subsequently stored under vacuum for later use in TiO_2_ decoration processes.

### 4.3 Synthesis of H:(Au@Cu_2_O/TiO_2_) Nanoparticles

The photocatalytic composite was synthesized through the sol‐gel mixing method. As illustrated in Figure S1, 10 mL of an ethanol‐based suspension was prepared by thoroughly mixing predetermined amounts of Au@Cu_2_O and TiO_2_ powders. To ensure homogeneous dispersion of the reactants, the suspension underwent ultrasonic treatment for 2 h at room temperature. Following this, the resulting green Au@Cu_2_O/TiO_2_ composite powders were recovered through repeated centrifugation and washing steps, then dried under vacuum conditions.

To enhance photocatalytic performance, a hydrogenation process was employed. Specifically, the Au@Cu_2_O/TiO_2_ composites were annealed at 200°C for 2 h in a saturated methanol vapor atmosphere. The observed color change from olive green to dark (as shown in Figure S1) confirmed the successful hydrogenation of the Au@Cu_2_O/TiO_2_ composites. For comparative analysis of the hydrogenation effect on photocatalytic hydrogen evolution, a control sample was also prepared by subjecting the Au@Cu_2_O/TiO_2_ composite to vacuum annealing at 200 °C in the absence of methanol. This untreated sample served as a reference to evaluate the performance of the hydrogenated composite, denoted as H:(Au@Cu_2_O/TiO_2_). Meanwhile, in order to clarify the role of oxygen vacancy in photocatalytic hydrogen evolution contribution, we had designed a series experiment without LSPR induced effect by Cu_2_O/TiO_2_ and H:(Cu_2_O/TiO_2_) catalysts. The photocatalysts were synthesized following the same preparation method as Au@Cu_2_O/TiO_2_ and H:(Au@Cu_2_O/TiO_2_) without adding Au colloids as a core.

### 4.4 Photocatalytic Hydrogen Evolution

This study focuses on quantifying hydrogen (H_2_) evolution during the photocatalytic process under AM 1.5 simulated sunlight, with methanol serving as an efficient sacrificial agent. In a typical experiment, 2–10 mg of photocatalyst powder was dispersed in 40 mL of a 20% v/v methanol aqueous solution, contained within a sealed square glass vessel capped with a rubber septum. Prior to illumination, the system was purged with argon gas for 15 min to eliminate residual oxygen and ensure an inert atmosphere. During the continuous AM 1.5 illumination, 1 mL of the headspace gas was extracted hourly via a syringe needle for analysis. The quantity of hydrogen produced (in moles) was determined by integrating the corresponding peak areas from gas chromatography–mass spectrometry (GC‐MS) measurements. The hydrogen evolution rate (HER) was calculated in mmol g^−^^1^ h^−^^1^ based on the mass of photocatalyst used in each experiment.

The apparent quantum yield (AQY) was evaluated under monochromatic light irradiation using band‐pass filters at specific wavelengths (λ = 380, 420, 560, 650, 750, 800, and 890 nm) in conjunction with AM 1.5 illumination. AQY was calculated by comparing the number of electrons generated (as inferred from evolved hydrogen) with the number of incident photons, according to the following equation [61]:

(10) $$\begin{array}{ccc} \mathrm{AQY}\left(\%\right) & = & \frac{\#\;\mathrm{of}\;\mathrm{ou}\mathrm{tcom}e\;\mathrm{ele}\mathrm{ctro}\mathrm{ns}}{\#\;\mathrm{of}\;\mathrm{in}\mathrm{cide}\mathrm{nt}\;\mathrm{ph}\mathrm{otons}}\times 100\%\\ & = & \frac{2\times \#\;\mathrm{of}\;\mathrm{hy}\mathrm{drog}\mathrm{en}\;\mathrm{mo}\mathrm{lecu}\mathrm{les}\;p\mathrm{rodu}\mathrm{ced}}{\#\;\mathrm{of}\;\mathrm{in}\mathrm{cide}\mathrm{nt}\;\mathrm{ph}\mathrm{otons}\left(\lambda \right)}\times 100\% \end{array}$$

### 4.5 SSA

The specific surface area was determined from N_2_ adsorption–desorption isotherms at 77 K using a Micromeritics ASAP 2020 analyzer, applying the Brunauer–Emmett–Teller (BET) and the Barrett–Joyner–Halenda (BJH) model for analysis. Samples were degassed at 120°C before measurement. The SSA of the react powders was obtained from 50 mg powders.

### 4.6 TAS

The femtosecond transient absorption spectra were obtained using an Exci Pro transient absorption spectrometer (CDP systems). The transient absorption system is equipped with an ultrashort pulse amplifier (Legend USP, Coherent, 795 nm, 3 mJ). The output from the amplifier is split to generate pump and probe pulses. The excitation wavelength was set to 397 nm by doubling the fundamental using a BBO crystal, whereas the probe pulses were generated using a white supercontinuum generated by pumping a 5 mm sapphire plate with a weak portion of the fundamental pulse. The excitation pulse energy was set to 250 nJ. The acquired spectra were corrected for the group velocity dispersion of the white light.

### 4.7 PEC

The transient photoelectrochemical measurements were done by using CHI611E electrochemical analyzer with a three‐electrode setup. The working electrode was a thin film of photocatalyst coated [62] on ITO glass with 3% polyvinylidene difluoride (PVDF) binder. In particular, 5mg of photocatalyst was mixed with 10µL PVDF solution prepared in N‐methyl‐2‐pyrrolidone (NMP) and sonicated for 2 min, and then drop‐cast on 1cm^2^ exposed area of ITO, while the remaining part of ITO was masked by Kapton tape. The films are then dried in a vacuum oven at 60°C for 12h before PEC measurements. To track the actual efficiency of these samples under AM1.5 illumination without applied bias, 20% methanol solution was used as electrolyte during PEC experiments. The light irradiation was on the ITO side of the thin film for the efficient dragging of photoinduced charge carriers to ITO and thereby transferred to the electrochemical system. The measurements were done under cathodic polarity under 0V bias, for which the positive photocurrent increase indicates electron transfer to ITO, while a more negative photocurrent value on light irradiation shows hole transport to ITO from the photocatalyst.

### 4.8 Sample Characterization

The surface morphology of each photocatalyst was examined using a scanning electron microscope (JEOL JSM‐6700F). Detailed crystallinity and microstructural features of the substrates were characterized via a spherical aberration–corrected transmission electron microscope (JEOL JEM‐ARM200F) operated at 200 kV. X‐ray diffraction (XRD) patterns were collected using a Bruker AXS GmbH D8 Advance diffractometer (Cu Kα, λ = 1.5418 Å). Optical absorption spectra (UV–Vis) were recorded with a Hitachi U‐3010 spectrophotometer. Raman spectra of TiO_2_, Au@Cu_2_O/TiO_2_, and H‐modified (Au@Cu_2_O/TiO_2_) samples were acquired using an Ar^+^‐ion laser (λ = 514.5 nm) for direct comparative analysis. High‐resolution X‐ray photoelectron spectra (HRXPS) were obtained via a ULVAC‐PHI Quantera II instrument. Finally, hydrogen evolution rates were measured under AM 1.5 solar illumination (Newport LCS‐100 solar simulator, filter 94011A), with gas‐phase products quantified by a Bruker SCION 436 gas chromatograph. The Ti L‐edge soft X‐ray absorption spectra (XAS) were measured using a surface‐sensitive total‐electron‐yield (TEY) detector at the TLS beamline 20A, achieving an energy resolution of approximately ΔE/E ∼ 1/5000. The XAS data of the photocatalysts were collected in situ under illumination from a 300 W Xe lamp equipped with an AM1.5G filter, as well as in dark conditions.

## Conflicts of Interest

The authors declare no conflicts of interest.

## Supporting information




**Supporting File**: smll72869‐sup‐0001‐SuppMat.docx.

## Data Availability

The data that support the findings of this study are available from the corresponding author upon reasonable request.
