# Supplementary material for: Unraveling Interband Hot‐Electron Transfer in Hydrogenated Au@Cu2O/TiO2 Heterostructure Nanocrystals for Enhanced Hydrogen Evolution
Source: Small. 2026 Feb 23;22(22):e11114. doi: 10.1002/smll.202511114 (PMC13089095; doi:10.1002/smll.202511114)
Supplement: Supplementary file 1 — Supporting File: smll72869‐sup‐0001‐SuppMat.docx. [file SMLL-22-e11114-s001.docx]

**Supporting Information**

**UNRAVELING INTERBAND HOT-ELECTRON TRANSFER IN HYDROGENATED Au@Cu_2_O/TiO_2_ HETEROSTRUCTURE NANOCRYSTALS FOR ENHANCED HYDROGEN EVOLUTION**

*Tsai-Te Wang^†^, Shan-Jen Yang^†^, Sudhakar Narra, Rohit R Koli, Yu-Ru Lin, Yi-Dong Lin, Eric Wei-Guang Diau,^*^ Yung-Jung Hsu,^,*^ Yan-Gu Lin,^*^ and Ming-Chang Lin^*^*

Tsai-Te Wang, Yu-Ru Lin, Yi-Dong Lin, Yan-Gu Lin

Scientific Research Division, National Synchrotron Radiation Research Center, Hsinchu 300092, Taiwan

**Shan-Jen Yang, Yan-Gu Lin, Yung-Jung Hsu**

Department of Materials Science and Engineering, National Yang Ming Chiao Tung University, Hsinchu 300093, Taiwan

**Sudhakar Narra, Rohit R Koli, Eric Wei-Guang Diau, Ming-Chang Lin**

Department of Applied Chemistry, National Yang Ming Chiao Tung University, Hsinchu 300093, Taiwan
**Eric Wei-Guang Diau, Ming-Chang Lin**

Center for Emergent Functional Matter Science, National Yang Ming Chiao Tung University, Hsinchu 300093, Taiwan

Corresponding author:

**Yan-Gu Lin** Email: lin.yg@nsrrc.org.tw

**Yung-Jung Hsu** Email: [yhsu@cc.nctu.edu.tw](mailto:yhsu@cc.nctu.edu.tw)

**Eric Wei-Guang Diau** Email: [diau@mail.nctu.edu.tw](mailto:diau@mail.nctu.edu.tw)

**Ming-Chang Lin** Email: [chemmcl@emory.edu](mailto:chemmcl@emory.edu)

*^†^*These authors contributed equally to this work.

The detailed experimental procedures of H:(Au@Cu_2_O/TiO_2_) preparation, XRD, UPS analyses and the optimization of H:(Au@Cu_2_O/TiO_2_) by controlling Au@Cu_2_O and TiO_2_ ratio are provided. Additional HER and AQY comparisons of results based on Au/TiO_2_ and Cu_2_O/TiO_2_ system were presented.

| **Table S1** XRD analysis for Au@Cu_2_O based photocatalysts. | | | | |
| --- | --- | --- | --- | --- |
| Sample | Cu_2_O (111) | | | Lattice constant |
|  | Xc (2θ) | FWHM | D (nm) | a=b =c (**Å**) |
| Au@Cu_2_O | 36.38 | 0.634 | 13.18 | 4.274 |
| Au@Cu_2_O/TiO_2_ | 36.45 | 0.546 | 15.31 | 4.266 |
| H:(Au@Cu_2_O/TiO_2_) | 36.44 | 0.670 | 12.48 | 4.267 |

**Table S2** A comparison of Au/TiO_2_ or Cu_2_O/TiO_2_ based photocatalysts for H_2_ evolution.

| **Photocatalyst** | **Reaction details** | **HER**  **(mmol h^-1^ g^-1^)** | **AQY**  **% ( λ_ex_)** | **Reference** |
| --- | --- | --- | --- | --- |
| **Au@TiO_2_-graphene** | 25 % methanol; 300 W Xe lamp, λ > 400 nm | 0.68 | 0.53 % (450 nm) 0.31 % (700 nm) | [1] |
| **Cu_2_O/TiO_2_** | 10 % methanol; 300 W Xe lamp, λ > 420 nm | 24.83 | - | [2] |
| **TiO_2_-Au-CdS** | 0.1 M Na_2_S, 0.1 M Na_2_SO_3_; 300 W Xe lamp, λ > 420 nm | 0.67 | - | [3] |
| **Cu_2_O/TiO_2_** | 5 % TEA; 300 W Xe lamp | 14.39 | 0.56 % (350 nm) 0.05 % (700 nm) | [4] |
| **Cu_2_O/TiO_2_** | 4 % methanol; 200 W Hg-Xe lamp, λ > 420 nm | 0.048 | 3.5 % (> 420 nm) | [5] |
| **Au/TiO_2_** | 20 % methanol; 300 W Xe lamp, λ > 365 nm | 2.92 | 8 % (350 nm) 0.25 % (550 nm) | [6] |
| **Cu_2_O/TiO_2_** | 25 % methanol; Xe lamp | 1.38 | - | [7] |
| **Cu_2_O/TiO_2_** | 2.5 M methanol; 125 W Hg lamp | 0.25 (UV+Vis)  0.06 (Vis) | 2.31 % (UV+Vis) 1.51 % (Vis) | [8] |
| **Au/TiO_2_** | 5 % glycerol; 450 W Xe lamp | 18 | - | [9] |
| **Au-BaO@TiO_2_/CdS** | 5 % ethanol; 100 W Xe lamp | 13.54 | 7.14 % (420 nm) | [10] |
| **CuO-Cu_2_O/TiO_2_** | 10 % methanol; 150 W Hg-Xe lamp | 2.86 | - | [11] |
| **Au/ZrO_x_-TiO_2_** | 70 % methanol; 500 W Xe lamp | 4.2 (UV) 2.1 (Vis) | 3.6 % (UV) 1.9 % (Vis) | [12] |
| **IL@Au@TiO_2_** | 10 % methanol; LED lamp, λ = 365, 405 nm | 0.08  0.31 | 28.3 % (365 nm)  22.9 % (405 nm) | [13] |
| **Au@TiO_2_/CdS** | 5 % ethanol; 100 W Hg lamp | 19.15 | 4.04 % (420 nm) | [14] |
| **AuTiO_2_** | 10 % methanol; Xe lamp | 47.89 (UV+Vis) 0.00045 (Vis) | - | [15] |
| **Au@TiO_2_** | 10% methanol; Xe lamp | 1.54 (UV+Vis)  0.96 (Vis) |  | [16] |
| **Cu_2_O/TiO_2_** | 10 % methanol; 300W Xe lamp | 0.28 |  | [17] |
| **Cu_2_O/TiO_2_** | 22.1 % ethanol; Xe lamp | 8.51; |  | [18] |
| **H:(Au@Cu_2_O/TiO_2_)** | 20 % methanol; 150 W Xe lamp | 9.29 | 15.6 % (340 nm) 2.50 % (650 nm) | This work |

| **Table S3** The Ti^3+^ and Ti^4+^ ratio comparison of TiO_2_, Au@Cu_2_O/TiO_2_ and H:( Au@Cu_2_O/TiO_2_). | | |
| --- | --- | --- |
|  | **Ti^3+^ (%)** | **Ti^4+^ (%)** |
| **TiO_2_** | **-** | **100** |
| **Au@Cu_2_O/TiO_2_** | **5.8** | **94.2** |
| **H:(Au@Cu_2_O/TiO_2_)** | **10.5** | **89.5** |


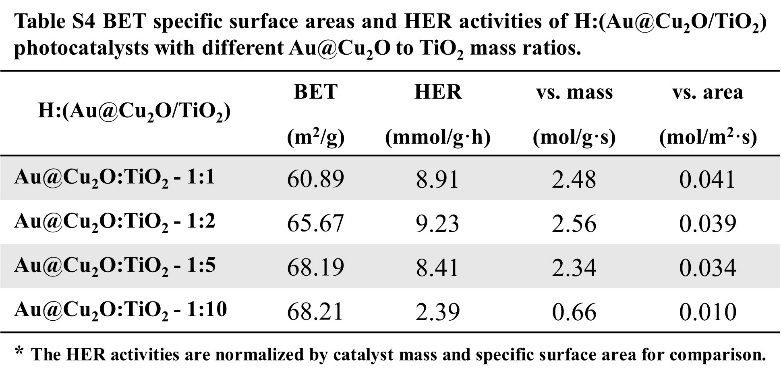


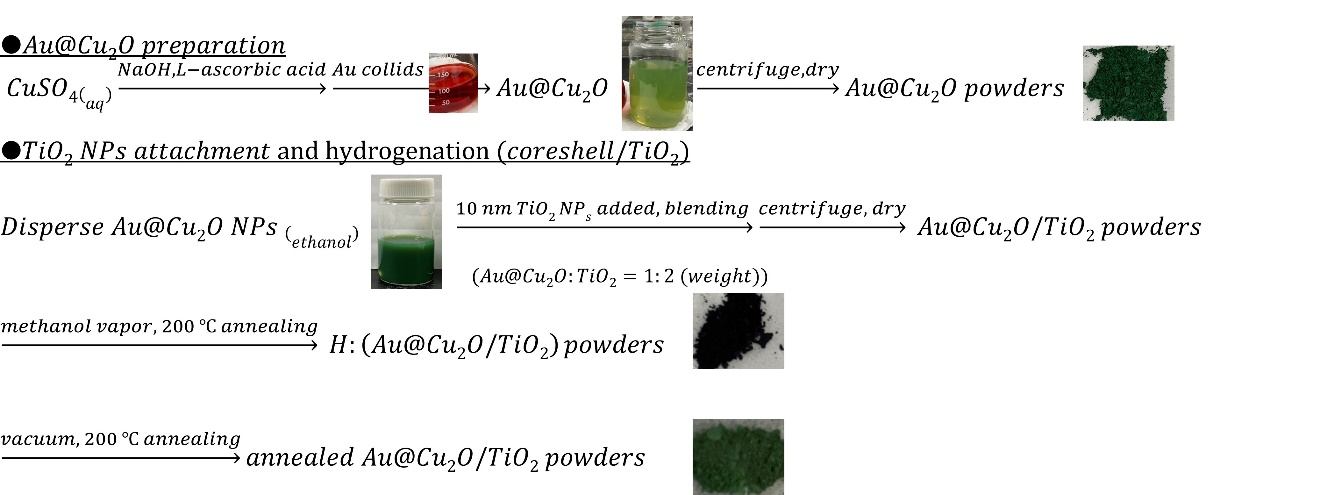


Figure S1. H:(Au@Cu_2_O/TiO_2_) preparation procedure.

The UV–Vis absorption spectra of Cu_2_O, Cu_2_O/TiO_2_, and H:(Cu_2_O/TiO_2_) are shown in Figure S2a. Pristine Cu_2_O exhibits strong absorption in the visible region with an absorption shoulder at 470 nm, consistent with its intrinsic band-gap absorption. A significant absorption band was observed in the UV range demonstrated the pristine TiO_2_ absorption by Cu_2_O/TiO_2_. Both Cu_2_O and Cu_2_O/TiO_2_ absorption terminate at 615 nm indicating the limitation of Cu_2_O application. The H:(Cu_2_O/TiO_2_) absorption extended through the visible range suggests the oxygen vacancy induced by hydrogenation. To distinguish the Cu material chemical’s state, the XPS spectrum was provided in Figure S2b. Pristine Cu_2_O shows characteristic Cu^+^ features without an obvious Cu^2+^ shake-up satellite, confirming the dominant Cu^+^ oxidation state. In contrast, Cu_2_O/TiO_2_ shows the coexistence of Cu^+^ and Cu^2+^ species, where peak fitting suggests that the Cu^2+^ component accounts for approximately 38% of the total Cu 2p signal, which can be attributed to interfacial interactions and partial surface oxidation. After hydrogenation, the relative contribution of Cu^2+^ species is clearly reduced to about 22%, indicating partial reduction of the surface Cu^2+^ species and the modification of the local electronic environment, consistent with defect formation during the hydrogenation process.


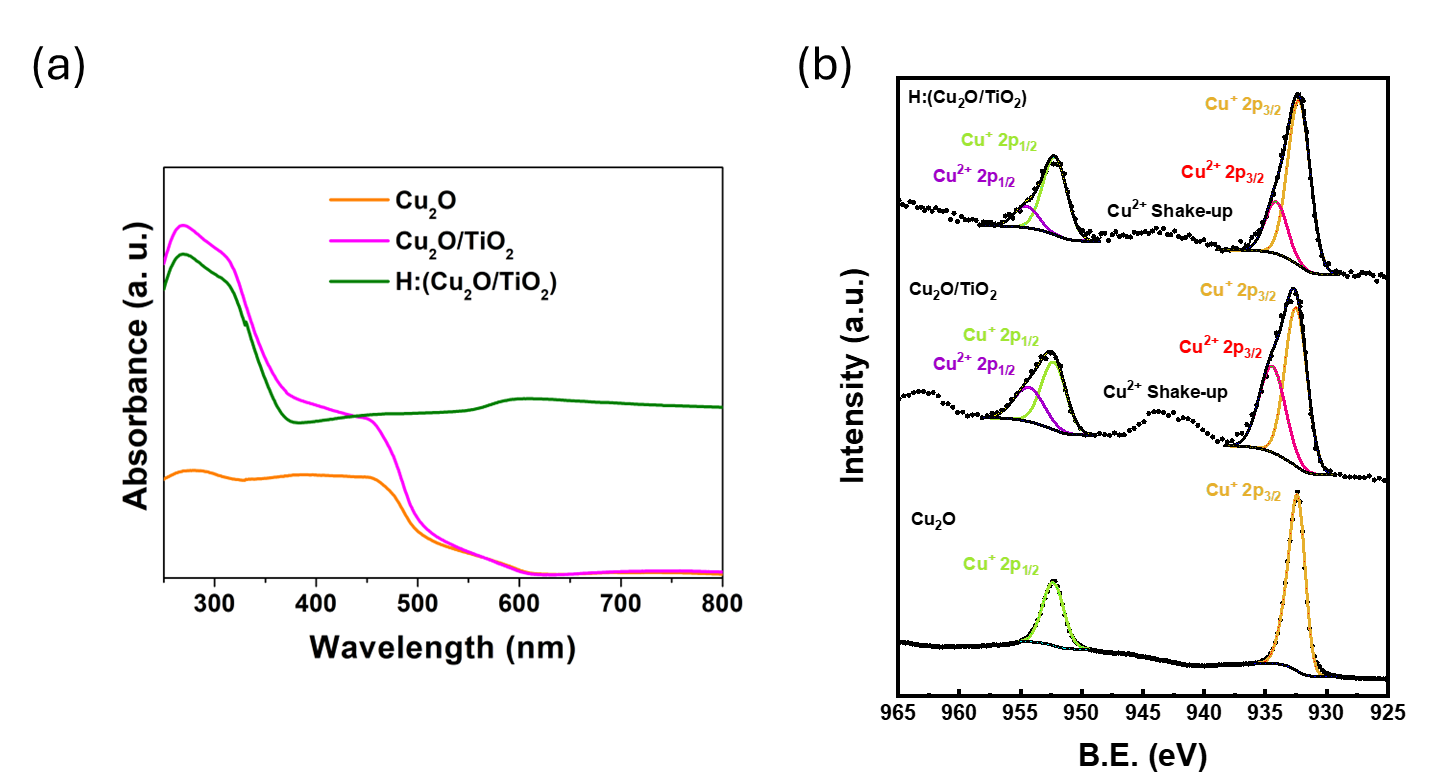


**Figure S2.** The (a) UV-Vis spectra, (b) Cu 2p XPS spectra of Cu_2_O, Cu_2_O/TiO_2_ and H:(Cu_2_O/TiO_2_).


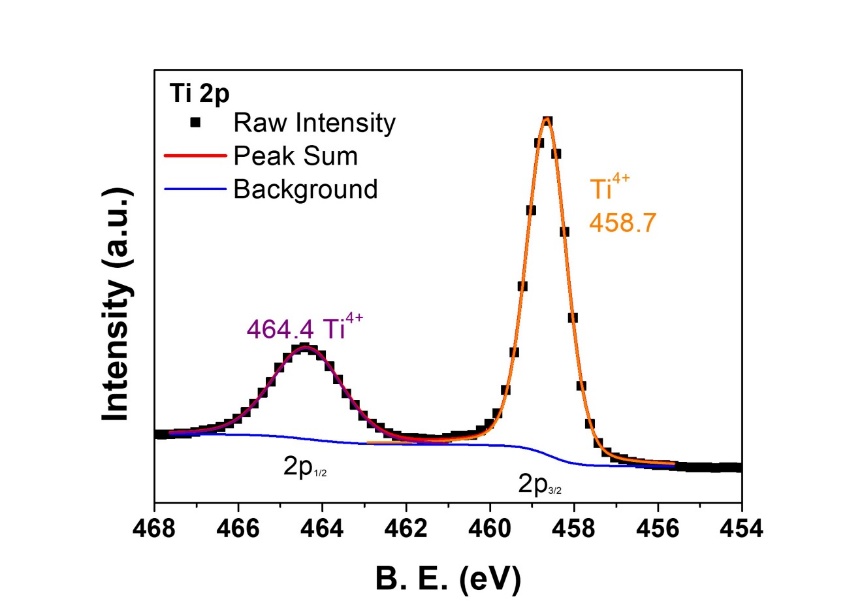


**Figure S3.** The Ti 2p xps spectroscopy of pristine TiO_2_.


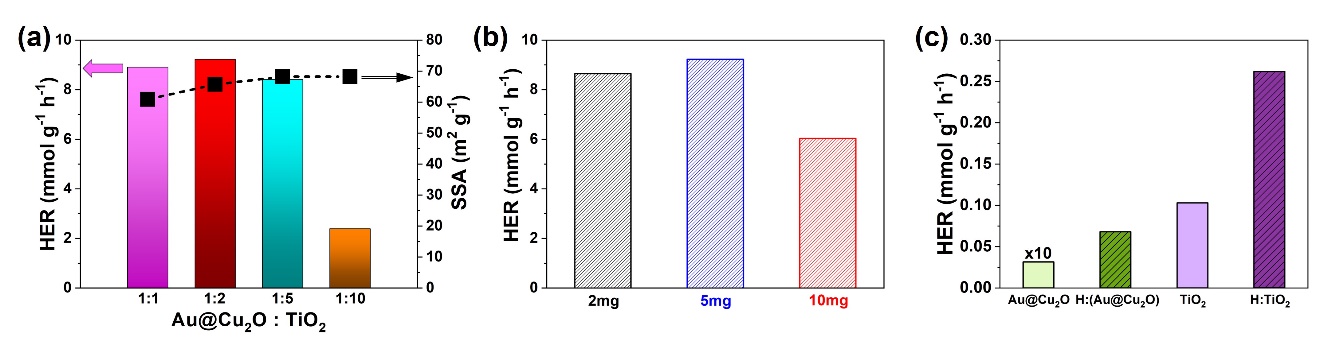


**Figure S4.** The bar charts of averaged HER (mmol g^-1^ h^-1^) results. a) varying Au@Cu_2_O: TiO_2_ ratio in photocatalysts (weight) from 1:10, 1:5, 1:2 to 1:1 with 5 mg photocatalysts H:(Au@Cu_2_O/TiO_2_) (bar chart) and SSA of 50 mg powders (scatter-line) used. b) 2, 5 and 10 mg H:(Au@Cu_2_O/TiO_2_) (1:2) sample. c) 5 mg Au@Cu_2_O, H:(Au@Cu_2_O), TiO_2_ and H:(TiO_2_) sample.
The HER bar charts were obtained from 5 h AM1.5 continuous illumination with 20% (v/v) methanol sacrificial solution. The SSA scatter-line was acquired from the Brunauer–Emmett–Teller (BET) and the Barrett–Joyner–Halenda (BJH) model for analysis.

**
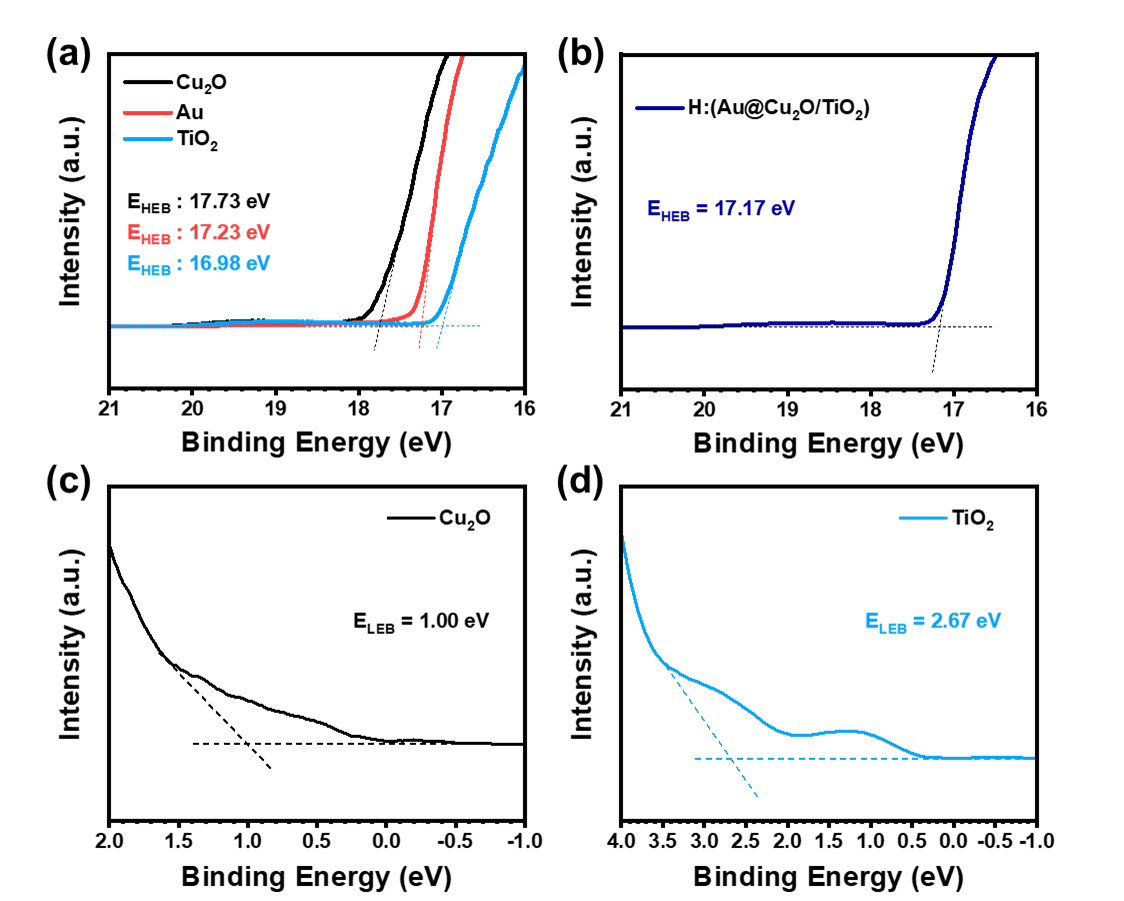
**
**Figure S5.** Analysis of UPS spectra a) pure Cu_2_O, pure Au and pure TiO_2_ b) H:(Au@Cu_2_O/TiO_2_) and valence band distribution c) pure Cu_2_O, d) pristine TiO_2_.

*UPS energy source: He I, $E_{h\upsilon}=21.2 eV$

**$E_{f}=E_{HEB}-E_{h\upsilon}; E_{VBM}=E_{f}-E_{LEB}$


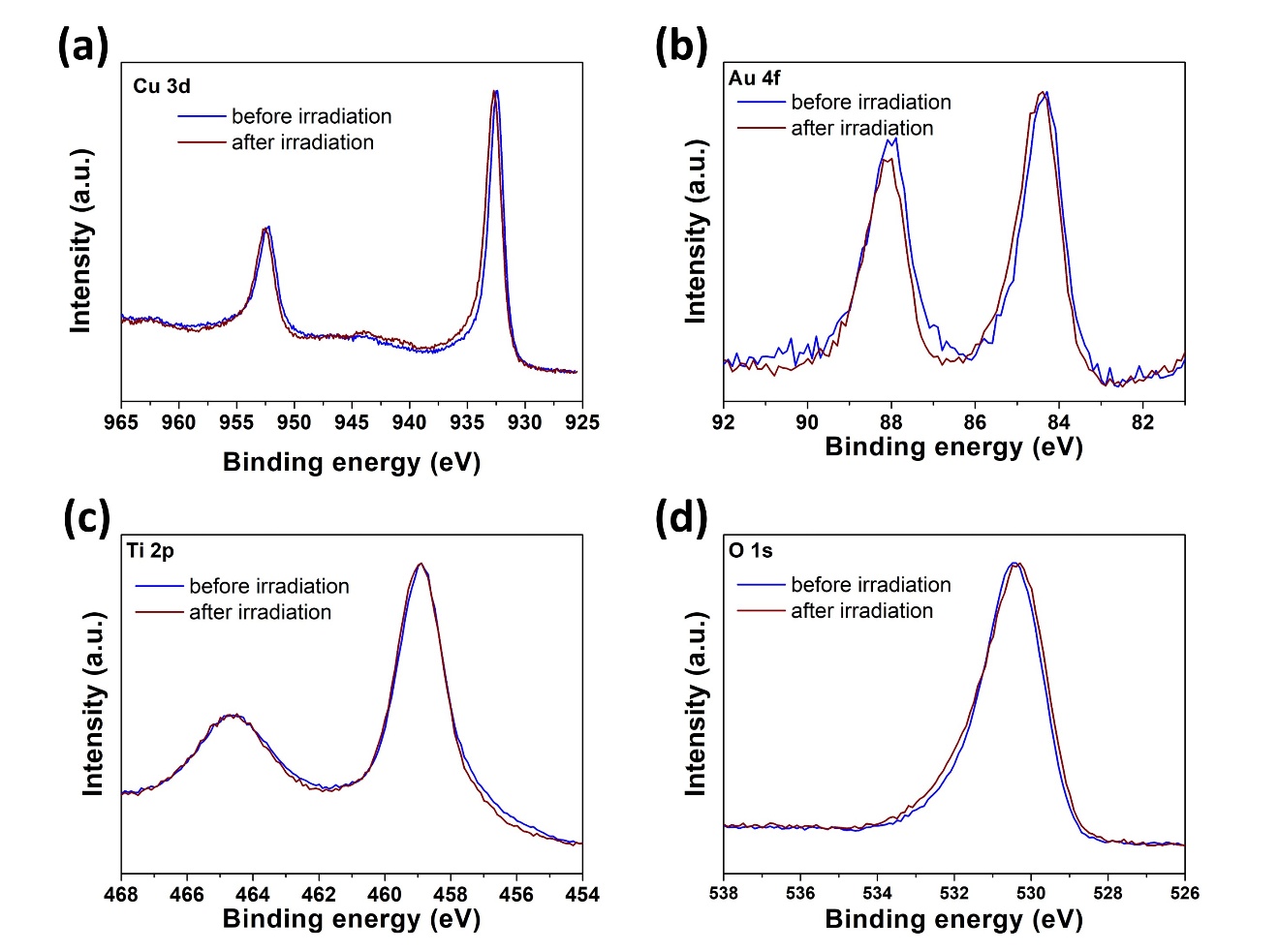


**Figure S6.** The XPS spectroscopy of H:(Au@Cu_2_O/TiO_2_) before and after irradiation.


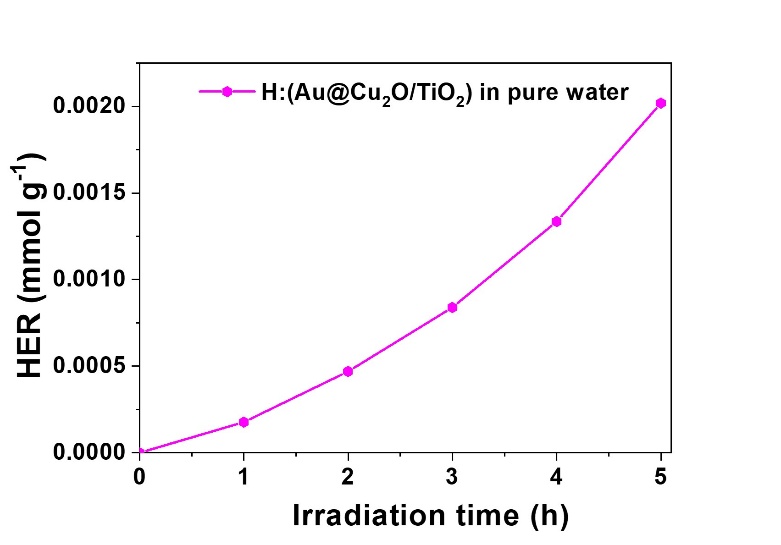


**Figure S7.** Hydrogen production activity of 5 mg photocatalysts H:(Au@Cu_2_O/TiO_2_) powders dispersed in pure water under 5 h AM1.5 illumination.

The transient photoelectrochemical measurements


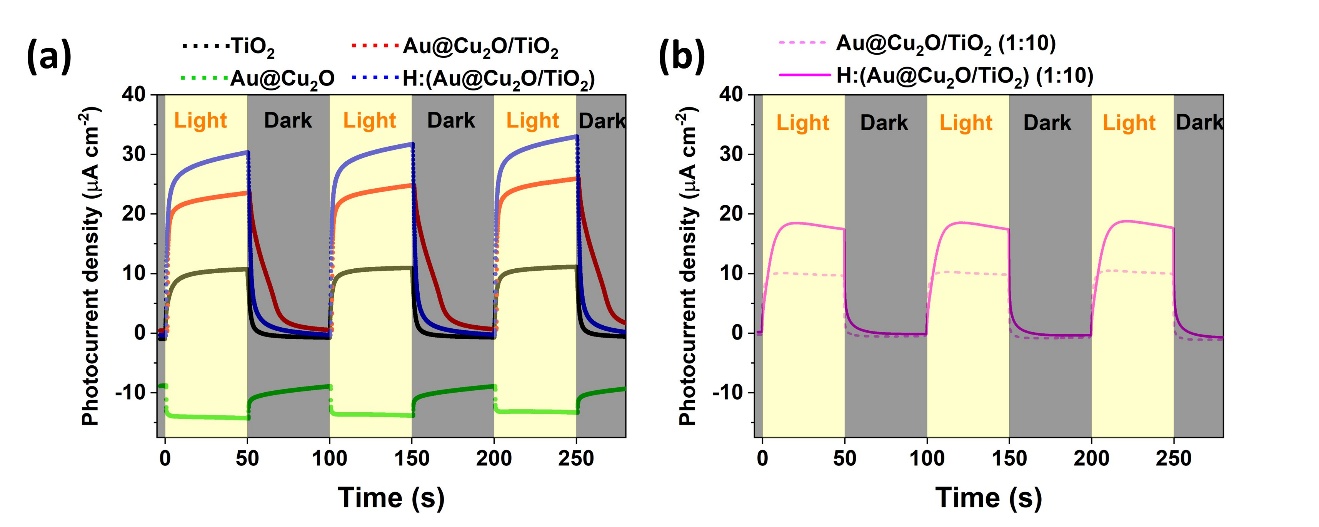


**Figure S8**. The PEC results with 3-electrode setup at 0 V bias voltage for a) TiO_2_, Au@Cu_2_O, Au@Cu_2_O/TiO_2_ (1:2), H:(Au@Cu_2_O/TiO_2_) (1:2) and b) Au@Cu_2_O/TiO_2_ (1:10), H:(Au@Cu_2_O/TiO_2_) (1:10). 5 mg sample was used with 3 wt% PVDF to coat on ITO glass. 1cm^2^ thinfilm substrate was prepared for photocurrent measurement.


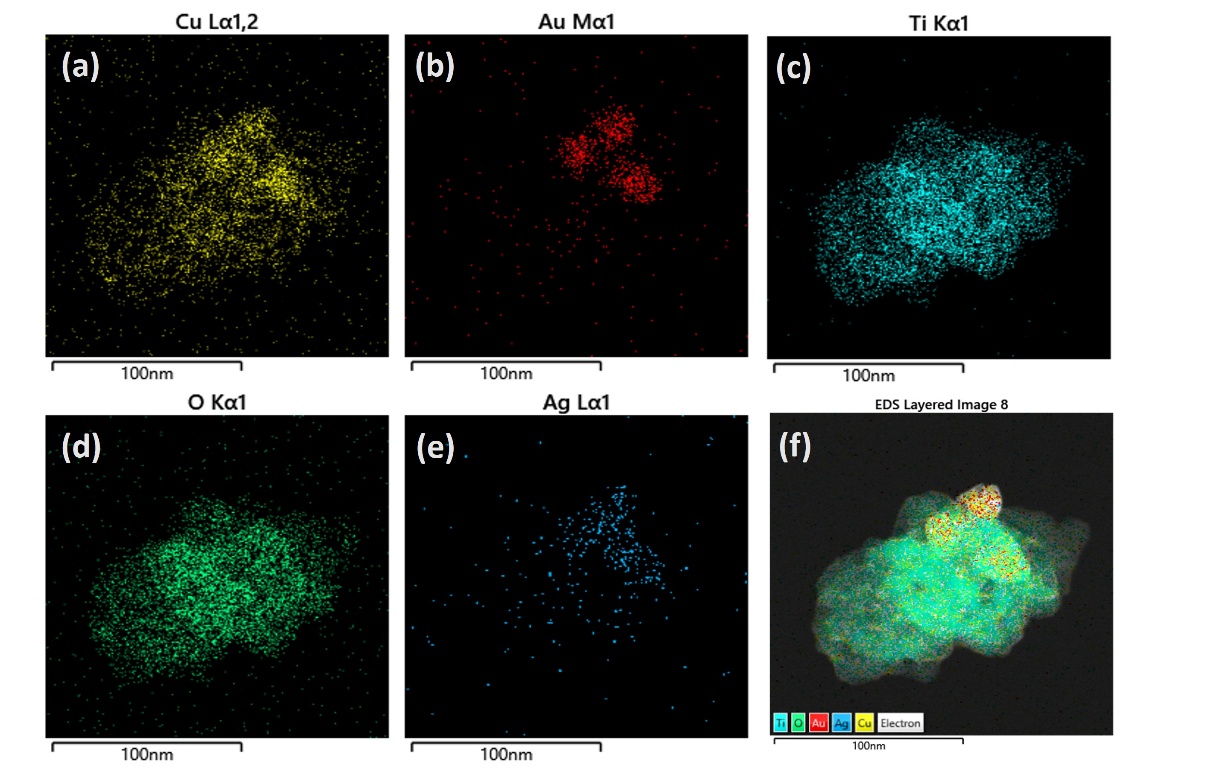


**Figure S9**. TEM image of Ag selectively photo-deposited on H:(Au@Cu_2_O/TiO_2_) composite. a) Cu, b) Au, c) Ti, d) O, e) Ag and f) combined results.


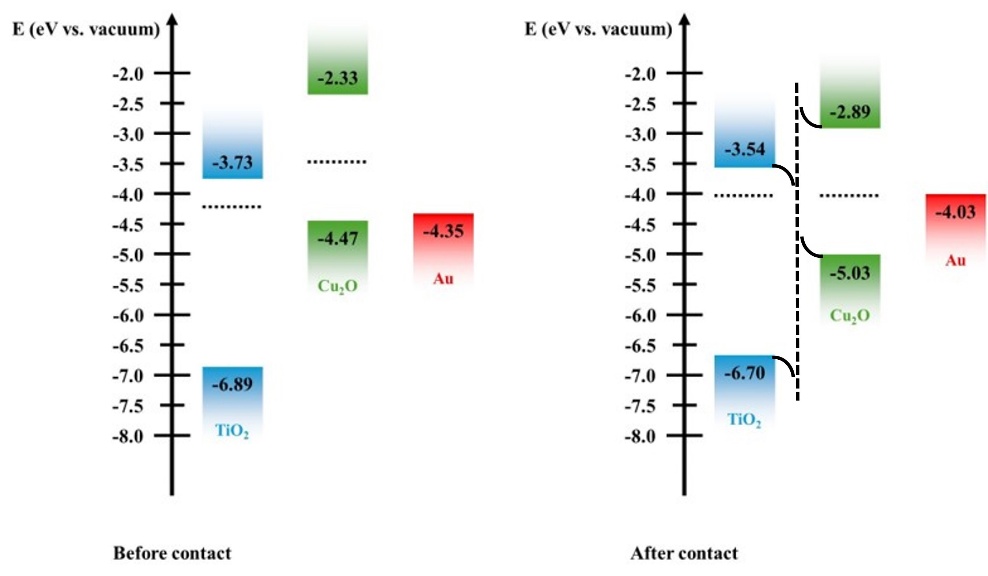


**Figure S10**. Schematic illustration of the energy band alignment before and after contact for H:(Au@Cu_2_O/TiO_2_).

***
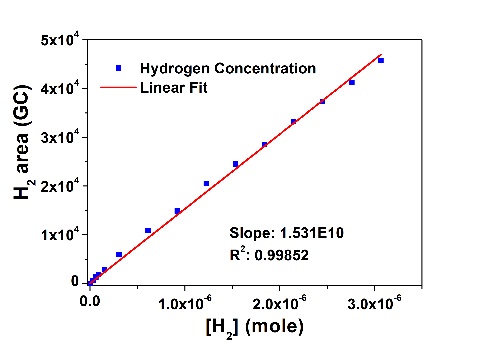
*Figure S11**. The H_2_ concentration calibration curve in GC detection.

**SI Reference**

1. H. Jung, J. Song, S. Lee, et al., “Hierarchical metal–semiconductor–graphene ternary heteronanostructures for plasmon-enhanced wide-range visible-light photocatalysis,” *Journal of Materials Chemistry A* 7 (2019): 15831-15840, <https://doi.org/10.1039/C9TA03934A>.
2. G. Li, J. Huang, J. Chen, et al., “Highly Active Photocatalyst of Cu_2_O/TiO_2_ Octahedron for Hydrogen Generation,” *ACS Omega* 4 *(*2019): 3392-3397, <https://doi.org/10.1021/acsomega.8b03404>.
3. X. Yao, X. Hu, W. Zhang, et al., “Mie resonance in hollow nanoshells of ternary TiO_2_-Au-CdS and enhanced photocatalytic hydrogen evolution,” *Applied Catalysis B: Environmental* 276 (2020): 119153, <https://doi.org/10.1016/j.apcatb.2020.119153>.
4. X. Bai, B. Liu, W. Zhang, et al., “MOF-derived porous TiO_2_ decorated with n-type Cu_2_O for efficient photocatalytic H_2_ evolution,” *New Journal of Chemistry* 45 (2021): 17332-17338, <https://doi.org/10.1039/D1NJ03089B>.
5. K. Sekar, C. Chuaicham, B. Vellaichamy, et al., “Cubic Cu_2_O nanoparticles decorated on TiO_2_ nanofiber heterostructure as an excellent synergistic photocatalyst for H_2_ production and sulfamethoxazole degradation,” *Applied Catalysis B: Environmental* 294 (2021): 120221, <https://doi.org/10.1016/j.apcatb.2021.120221>.
6. E. Lu, Z. Zhang, J. Tao, Z. Yu, Y. Hou and J. Zhang, “Enhanced Metal–Semiconductor Interaction for Photocatalytic Hydrogen-Evolution Reaction,” *Chemistry – A European Journal* 28 no.56 (2022): e202201590, <https://doi.org/10.1002/chem.202201590>.
7. J. L. Chen, M. M. Liu, S. Y. Xie, et al., “Cu_2_O-loaded TiO_2_ heterojunction composites for enhanced photocatalytic H_2_ production,” *Journal of Molecular Structure* 1247 (2022): 131294, <https://doi.org/10.1016/j.molstruc.2021.131294>.
8. M. Muscetta, S. A. Jitan, G. Palmisano, et al., “Visible light – driven photocatalytic hydrogen production using Cu_2_O/TiO_2_ composites prepared by facile mechanochemical synthesis,” *Journal of Environmental Chemical Engineering* 10 no.3 (2022): 107735, <https://doi.org/10.1016/j.jece.2022.107735>.
9. H. Bajpai, I. Chauhan, K. N. Salgaonkar, N. B. Mhamane, C. S. Gopinath, “Biomass components toward H_2_ and value-added products by sunlight-driven photocatalysis with electronically integrated Au^δ−^–TiO_2_: concurrent utilization of electrons and holes,” *RSC Sustainability* 1 (2023): 481-493, <https://doi.org/10.1039/D2SU00145D>.
10. M. Sabir, K. Rafiq, M. Z. Abid, et al., “Growth of tunable Au-BaO@TiO_2_/CdS heterostructures: Acceleration of hydrogen evolution from water splitting,” *Fuel* 253 (2023): 129196, <https://doi.org/10.1016/j.fuel.2023.129196>.
11. F. Plascencia-Hernández, E. Albiter, G. M. Nawfal, et al., “Unraveling the effect of low Cu_2_O loading on P25 TiO_2_ and its self-reduction during methanol photoreforming, *Inorganic Chemistry Communications* 158 no.1 (2023): 111541, <https://doi.org/10.1016/j.inoche.2023.111541>.
12. L. A. Arce-Saldaña, U. Caudillo-Flores, R. Sayago-Carro, et al., “Hydrogen photoproduction using Au promoted ZrO_x_-TiO_2_ composite catalysts,” *Catalysis Today* 419 no.1 (2023): 114148, <https://doi.org/10.1016/j.cattod.2023.114148>.
13. R. G. Chacón, F. S. Rodembusch, B. L. Albuquerque, et al., “Photocatalytic effects on Au@TiO_2_ confined in BMIm.NTf_2_ ionic liquid for hydrogen evolution reactions,” *International Journal of Hydrogen Energy* 48 no.81 (2023): 31629-31642, <https://doi.org/10.1016/j.ijhydene.2023.04.243>.
14. K. Rafiq, M. Sabir,M. Z. Abid, et al., “Tuning of TiO_2_/CdS Hybrid Semiconductor with Au Cocatalysts: State-of-the-Art Design for Sunlight-Driven H_2_ Generation from Water Splitting,” *Energy & Fuels* 38 no.5 (2024): 4625-4636, <https://doi.org/10.1021/acs.energyfuels.3c04785>.
15. C. Ban, B. Li, J. Ma, et al., “Plasmonic Au–TiO_2_ interactions for augmented photocatalytic hydrogen evolution,” *Ceramics International* 50 no.9 (2024): 15444-15451, <https://doi.org/10.1016/j.ceramint.2024.02.020>.
16. R. Ninakanti, R. Borah, T. Craig, et al., “Au@TiO_2_ Core–Shell Nanoparticles with Nanometer-Controlled Shell Thickness for Balancing Stability and Field Enhancement in Plasmon-Enhanced Photocatalysis,” *ACS Nano* 18 no.49 (2024): 33430-33440, <https://doi.org/10.1021/acsnano.4c09944>.
17. H. Qian, B. Yuan, Y. Liu, L. Wang, R. Zhu and P. Dong, “Effect of Cu valence states on conduction band position and reduction selectivity of TiO_2_-based heterojunction photocatalysts,” *iScience* 28 no.6 (2025): 112697, <https://doi.org/10.1016/j.isci.2025.112697>.
18. E. d. Couto-Pessanha, V. M. Paiva, M. D. R. Henriques, et al., “TiO_2_/Cu_2_O Heterojunction with Ultrafine Cu_2_O Dispersion and Enhanced Performance for Solar-Driven Hydrogen Production: A Low-Temperature, Ambient Pressure, and Common Stabilizing Agent-Free Synthesis Approach,” *ACS Omega* 10 no.35 (2025): 39814-39822, <https://doi.org/10.1021/acsomega.5c03805>.
